# Mapping Calcium Dynamics in the Heart of Zebrafish Embryos with Ratiometric Genetically Encoded Calcium Indicators

**DOI:** 10.3390/ijms21186610

**Published:** 2020-09-10

**Authors:** Jussep Salgado-Almario, Manuel Vicente, Pierre Vincent, Beatriz Domingo, Juan Llopis

**Affiliations:** 1 Physiology and Cell Dynamics, Centro Regional de Investigaciones Biomédicas (CRIB), Facultad de Medicina de Albacete, Departamento de Ciencias Médicas, Universidad de Castilla-La Mancha, C/Almansa 14, 02006 Albacete, Spain; Jussep.Salgado@uclm.es (J.S.-A.); Manuel.Vicente@uclm.es (M.V.); 2 UMR8256, Biological Adaptation and Ageing, CNRS, Sorbonne Université, F-75005 Paris, France

**Keywords:** calcium, genetically encoded calcium indicator (GECI), biosensor, zebrafish, embryo, heart, imaging

## Abstract

Zebrafish embryos have been proposed as a cost-effective vertebrate model to study heart function. Many fluorescent genetically encoded Ca^2+^ indicators (GECIs) have been developed, but those with ratiometric readout seem more appropriate to image a moving organ such as the heart. Four ratiometric GECIs based on troponin C, TN-XXL, Twitch-1, Twitch-2B, and Twitch-4 were expressed transiently in the heart of zebrafish embryos. Their emission ratio reported the Ca^2+^ levels in both the atrium and the ventricle. We measured several kinetic parameters of the Ca^2+^ transients: systolic and diastolic ratio, the amplitude of the systolic Ca^2+^ rise, the heart rate, as well as the rise and decay times and slopes. The systolic ratio change decreased in cells expressing high biosensor concentration, possibly caused by Ca^2+^ buffering. The GECIs were able to report the effect of nifedipine and propranolol on the heart, which resulted in changes in heart rate, diastolic and systolic Ca^2+^ levels, and Ca^2+^ kinetics. As a result, Twitch-1 and Twitch-4 (*K*d 0.25 and 2.8 µM, respectively) seem the most promising GECIs for generating transgenic zebrafish lines, which could be used for modeling heart disorders, for drug screening, and for cardiotoxicity assessment during drug development.

## 1. Introduction

Many genetic and acquired heart diseases are believed to involve a dysregulation of the Ca^2+^ signaling [1,2,3]. Since the Ca^2+^ ion is central in excitation–contraction coupling in the heart, genetically encoded Ca^2+^ indicators (GECIs) have been used in animal models to image the physiological and pathophysiological mechanisms and to characterize the actions of drugs on the heart [4]. Compared with studies in isolated cardiomyocytes, animal studies have the advantage of keeping the relationship between the heart and other organs. The heart is monitored while performing mechanical work and driving blood flow in a physiological environment. Thus, molecular or cellular changes can be correlated to important functional outcomes, such as cardiac output or ejection fraction.

The zebrafish embryo is emerging as a cost-effective, relatively easy-to-study specimen, because of its high genetic manipulability, transparency, ex-utero embryonic development, and large number of offspring. The zebrafish heart is composed of an atrium and a ventricle, with many anatomical differences with the mammalian heart. However, it has been argued that the zebrafish heart physiology, heart rate (HR), and action potential, while showing some channel expression differences, are more similar to those of the human than other animal models, such as the mouse [5,6,7,8,9,10,11]. For all these reasons, the zebrafish is increasingly considered a model in cardiovascular research in phenotype-based drug and genetic screenings [12,13,14,15]. However, some concerns have been pointed out regarding their use as models for human heart disease [16,17]. Reported differences with the mammalian heart are a small contribution of the sarcoplasmic reticulum to the Ca^2+^ transient, lack of T-tubules and lower expression of ryanodine receptors [11,16], or a larger density of Ca^2+^ current through the L-type Ca^2+^ channels (LTCC) and Ca^2+^ entry through the Na–Ca exchanger (NCX, at membrane potentials above +10 mV) [11,17]. These have led some authors to caution about the extrapolation of the functional impact of mutations studied in zebrafish to the human [11,17]. We believe that further studies are needed to characterize the Ca^2+^ handling in embryonic, larval, and adult zebrafish cardiomyocytes, to find out which channel isoforms and transporters are common or differ from neonates and adults in the human [11] and, thus, which diseases can be modeled in the zebrafish. This emphasizes the importance of electrophysiological and imaging studies like this one. Moreover, molecular techniques like CRISPR/Cas9 will allow removal or introduction of wild type or mutated genes to generate zebrafish models more similar to the human heart [11].

Numerous GECIs have been developed and improved in recent years. They can be broadly classed as single-fluorophore GECIs, like the widely used GCaMPs [18,19,20,21], and ratiometric GECIs, bearing two fluorophores [22,23,24]. Imaging the former is technically simpler and allows the collection of all photons, whereas in ratiometric emission GECIs some photons are lost by using bandpass filters and beamsplitters. GCaMPs are frequently used in neuroscience and in cardiovascular research [18,23,25,26]. Recent papers have successfully used GCaMP6f [25] or GCaMP5G [26] in the heart of zebrafish embryos. These biosensors worked fine, and the authors measured the effect of channel blockers and adrenergic modulators on the embryo heart. We chose ratiometric GECIs to image Ca^2+^ in the heart of zebrafish embryos because the ratio should, in principle, largely cancel out motion artifacts, correct for differences in expression levels, specimen thickness, tissue growth, and illumination inhomogeneities [22,23,27,28]. In addition, ratiometric probes are usually brighter than many single fluorophore biosensors at resting Ca^2+^ levels.

Chemical and gene-encoded Ca^2+^ indicators aim to measure the free Ca^2+^ concentration because it represents its propensity to drive Ca^2+^-binding reactions, its chemical potential [29]. To do so, Ca^2+^ indicators react with that same Ca^2+^, acting as exogenous buffers and altering the Ca^2+^ dynamics and possibly Ca^2+^ function as well, depending on the biosensor mobility, affinity, and concentration [23,29,30,31]. Biosensor expression levels should be as low as possible while providing an adequate optical signal-to-noise ratio (SNR). At higher expression levels, buffering by the biosensor might unnecessarily interfere with the biological effects. A consequence of Ca^2+^ buffering is blunting of the transient increases, which is worse the higher the concentration of the exogenous buffer, the closer the intracellular Ca^2+^ to the *K*d of the biosensor, and the shorter the Ca^2+^ transients [29]. A second consequence is blurring of the spatial gradients, depending on the size and mobility of the Ca^2+^-bound biosensor compared to the endogenous buffers. Depression of the resting Ca^2+^ levels is not usually a concern in the presence of extracellular Ca^2+^ because low levels increase the Ca^2+^ entry mechanisms and slow down the Ca^2+^ efflux [29,32].

We chose the fluorescence resonance energy transfer (FRET)-based GECIs TN-XXL, Twitch-1, Twitch-2B, and Twitch-4, with in vitro properties, which a priori seemed adequate to work well in the embryo heart. The Twitch series of biosensors possess minimal Ca^2+^-binding domains and respond with a Hill slope close to 1, which improves the linearity of the fluorescence response with Ca^2+^ [23,24]. In turn, TN-XXL [33] has already been shown to monitor the Ca^2+^ changes in the mouse heart ex vivo [34]. The biosensors should be expressed at sufficient levels to be imaged at a high speed and, since in vitro conditions differ considerably from the intracellular milieu, their performance in vivo should be investigated empirically. This is important to fill a gap between the “proof-of-principle” demonstration of the performance of GECIs and their practical implementation as scientific tools, particularly in vivo. The Twitch biosensors we employed have been characterized mostly in the brain but to our knowledge they have never been used in the heart of any vertebrate in vivo. There are striking differences between the applications in the brain an in the heart. For use in the zebrafish heart, a major problem is the constant cycling between diastolic and systolic Ca^2+^ levels at a rate of about 200 bpm (more than 3 beats/s).

The TN-XXL and Twitch GECIs, composed of chicken or *Opsanus* troponin C as their Ca^2+^-sensing element, respectively, were found to interfere less with the cellular regulatory elements than the calmodulin-based biosensors (cameleon, GCaMPs) in the neurons [35]. However, in addition to Ca^2+^ buffering, the expression of biosensors containing troponin C could have deleterious effects on zebrafish heart physiology. They might hamper the excitation–contraction coupling in the myofibrils or affect the parameters such as the force of contraction. We investigated these aspects, sometimes overlooked in studies employing genetically encoded indicators. Direnberger et al. examined the off-target effects of TN-XXL expression in a transgenic mouse model [34].

The four GECI mentioned above were compared in 3 days post-fertilization (dpf) embryos transiently expressing the biosensors, to identify their particular advantages and limitations in the zebrafish heart. They were bright enough to be imaged at 50 or even 100 Hz, which was needed to resolve the Ca^2+^ changes in the heart occurring at >200 beats per minute (bpm). The GECIs were found to reliably report the Ca^2+^ changes during systole and diastole and to correct for motion artifacts and photobleaching. The biosensors showed a decrease in the Ca^2+^ levels and HR induced by a Ca^2+^ channel antagonist and an adrenergic blocker. In addition, this work allowed us to assess which GECIs would perform better in transgenic models. While Twitch-4 could be a good candidate for various applications because of its sensitivity and faster fluorescence response kinetics, the higher affinity Twitch-1 might be preferred to measure the diastolic or decreased Ca^2+^ levels associated with drugs or disease. Several recent papers have already demonstrated that zebrafish can be used for screening [13] and for detecting the deleterious effects of drugs on the heart in zebrafish embryos [14,15]. These studies were performed with transmitted light or in zebrafish lines expressing GFP in the heart, which allowed localization and measurement of the heart rate and other parameters. By expressing a Ca^2+^ biosensor, our approach provides both localization of the heart (by fluorescence) and a readout of an important second messenger (Ca^2+^).

## 2. Results

### 2.1. Transient Expression of TN-XXL and Twitch Ca^2+^ Biosensors in the Heart of Zebrafish Embryos

We selected four ratiometric GECIs to measure the in vivo Ca^2+^ changes in the heart of 3 dpf zebrafish embryos. The probes TN-XXL, Twitch-1, Twitch-2B, and Twitch-4, with different Ca^2+^ affinities and response kinetics, were chosen to characterize and compare their usefulness as Ca^2+^ reporters in the beating heart; Table 1 summarizes their in vitro properties. TN-XXL belongs to a family of GECIs with chicken troponin C as the Ca^2+^-binding domain [33]. It contains the C-terminal domain of the chicken skeletal muscle troponin C and was engineered to increase Ca^2+^ affinity (0.8 µM *K*d) and to block Mg^2+^ binding. Although it has been superseded by more recent biosensors, TN-XXL was chosen because it has been shown to report Ca^2+^ transients in beating sinoatrial node explants and dissected hearts from transgenic mice [34].

The Twitch biosensors are improved versions of TN-XXL with a reduced number of Ca^2+^-binding sites per biosensor and are based on the C-terminal domain of the toadfish *Opsanus tau* troponin C [24]. Twitch-1 and Twitch-2B have a high Ca^2+^ affinity (0.25 and 0.2 µM *K*d, respectively) and decay times of 0.8 s and 2.8 s. The biosensor with the lowest Ca^2+^ affinity, Twitch-4 (2.8 µM *K*d), possesses only one functional EF hand (Hill slope of 1.04) and has the fastest decay time constant of the employed GECIs (0.5 s) (Table 1). All biosensors contain the fluorescent proteins (FP) CFP as the FRET donor at the N-terminal end, and cpCitrine174, a YFP, as the FRET acceptor at the C-terminal end, except for Twitch-2B, which incorporates mCerulean3 as the donor and cpVenusCD as the acceptor.

**Figure 1:**
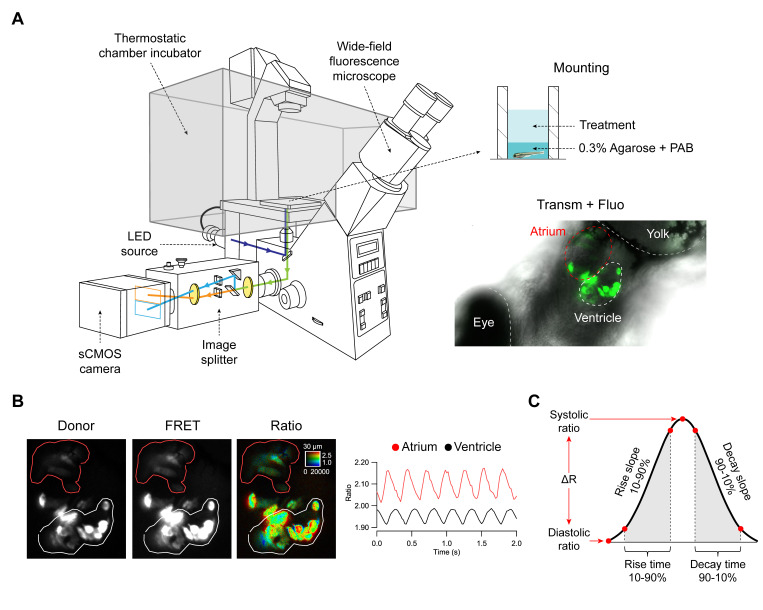
Zebrafish embryo mounting for microscopy, image acquisition and processing, and parameters extracted from Ca^2+^ transients. (**A**) Embryos expressing the biosensors in the heart were embedded in agarose and mounted in glass-bottom 96-well plates. An overlay of transmitted light and fluorescence of the heart is shown in an embryo expressing Twitch-4. The fluorescence from the widefield microscope passed through an image splitter that separated the FRET and donor emission onto the sCMOS sensor. (**B**) The two emission images were divided off-line and the ratio images (FRET/donor channels) were computed. Regions-of-interest (ROI) were manually drawn on the atrium (red line) and ventricle (white line) and analyzed in Igor Pro (WaveMetrics). (**C**) Various parameters were automatically extracted from the ratio time course data.

The coding sequences of TN-XXL, Twitch-1, Twitch-2B, and Twitch-4 were subcloned into the pTol expression vector [36,37] under the *cmlc2* (*myl7*) promoter [38], which drives expression of the probes in the atrium and ventricle of the zebrafish heart. To obtain embryos with transient expression, fertilized zebrafish eggs were microinjected with the cDNA of each biosensor and transposase mRNA. Embryos showed fluorescence in the forming cardiac tube at 24 h post-fertilization (hpf) and, by 3 dpf, all biosensors were expressed specifically in the atrium and ventricle (Figure 1A). Embryos were embedded in agarose on a 96-well plate and placed on the stage of a widefield fluorescence microscope fitted with an emission splitter, which projected CFP and YFP emission simultaneously on the sCMOS camera in order to obtain perfect time registration of the donor and FRET fluorescence (Figure 1A). As the HR was found to be very temperature-sensitive, embryos were imaged in a thermostatic chamber at 28 °C.

As expected, mosaic expression of the biosensors was seen in the heart and no other organ appeared fluorescent, except for autofluorescence of the yolk. Some cells were brightly fluorescent in both the CFP and FRET channels and the ventricles were more stained than the atria, as shown in Figure 1B for Twitch-4, owing in part to their thickness. A custom written program was used to compute the pseudo color ratio images of the FRET acceptor to donor fluorescence, where the hue coded for the ratio and brightness was proportional to the fluorescence intensity, so that no arbitrary thresholding of intensity was needed to identify the cells [28,39]. Since the HR of the 3 dpf embryos was above 200 bpm, they were imaged at 50 Hz with continuous illumination during 5–10 s to record the ratio changes in the beating hearts over time. To minimize the motion artifacts, the embryos were incubated with the non-fluorescent myosin inhibitor para-amino blebbistatin (PAB) [40], which decreased but did not completely abolish the heart contractions (compare Videos S1 and S2 in transmitted light and Videos S3–S5 for emission ratio of the different GECIs). Because of their small size, embryos at this stage can survive without heart contraction due to oxygen diffusion through the tissues [41]. Regions of interest (ROI) were manually drawn on the atrium and ventricle and their spatially averaged ratio values (related to Ca^2+^) were computed over time (Figure 1B).

Ratio levels were seen to oscillate synchronously with the heart contractions and the ratio rise started in the atrium and was propagated to the ventricle with a delay (Figure 1B; Videos S3 and S4). Zebrafish are known to have a functional pacemaker in the sinoatrial node, similarly to mammals. Although the existence of an atrioventricular node has not been established, there is a delay in the electrical conduction in the atrioventricular canal, and thus the ventricle contracts after filling with blood from the atrium.

We wrote routines in Igor Pro 8 (WaveMetrics) to analyze the ratio recordings (see Materials and Methods for details). A smoothing algorithm was applied to the raw ratio data (see Methods and Table S3). The routines automatically identified the peak and lowest ratio (R_systole_ and R_diastole_) in each cardiac cycle and calculated the following parameters: the Ca^2+^ transient amplitude ΔR (systolic minus diastolic ratio), the rate of Ca^2+^ rise and decrease (ratio rise slope from 10 to 90% and decay slope from 90 to 10% of the amplitude), the rise time 10–90%, and the decay time 90–10% of the Ca^2+^ transients (Figure 1C). From the time between systolic peaks, the instantaneous HR was also calculated.

**Figure 2:**
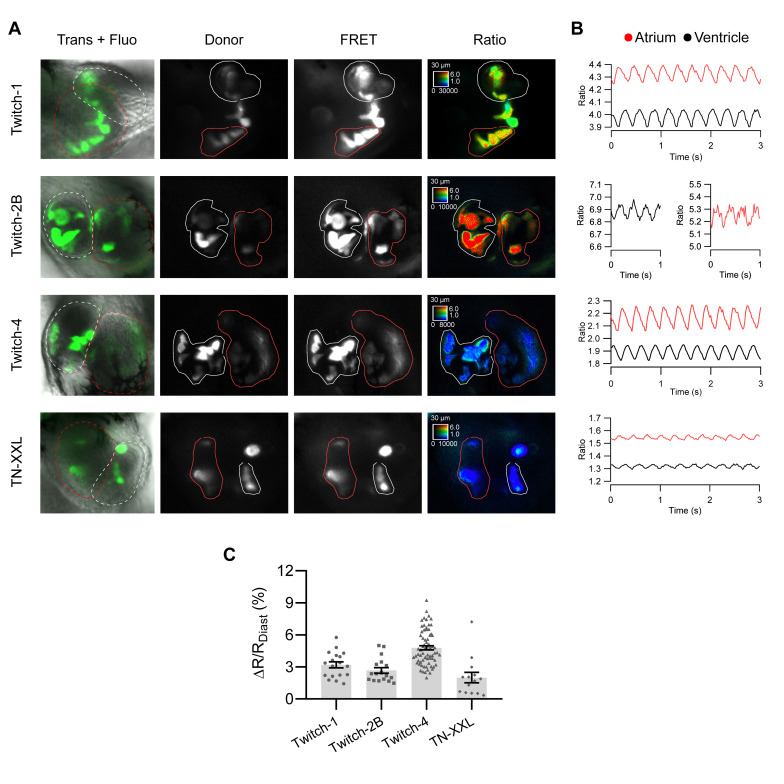
Ratiometric imaging of zebrafish heart Ca^2+^ dynamics with TnC-based biosensors. (**A**) Images of 3 dpf embryos transiently expressing the indicated biosensors at various levels in different cells owing to mosaicism. The overlay of transmission and fluorescence images show the location of the atrium and the ventricle. Donor and FRET channel images correspond to the emission of the donor and acceptor FP, both excited at 440 nm. The ROIs outlined in red and white were used to quantify changes in the atrium and ventricle, respectively. The emission ratio image (FRET image/donor image) is shown in pseudo color with the same scaling applied to all biosensors to facilitate their comparison. The calibration squares show the distance in µm, whereas the hue codes for the emission ratio and intensity codes for the fluorescence intensity. Embryos were treated with PAB to decrease the heart motion. (**B**) The traces of the emission ratio over time (acquired at 50 images/s) of the embryos displayed in (**A**) is shown (unprocessed data). The ratios were seen to oscillate in synchrony with heart contractions in the atrium (red) and ventricle (black). (**C**) Percentage of ratio change between systole and diastole (%(ΔR/R_diastole_)) obtained with each biosensor. The bars show the mean ± S.E.M, and each dot is one embryo, *n* = 14 to 70 embryos of one or four independent experiments.

Figure 2A shows the fluorescence of TN-XXL, Twitch-1, Twitch-2B, and Twitch-4 overlaid with the transmitted light in the representative experiments, as well as the donor and FRET fluorescence images and their ratio (in pseudo color with the same scaling). The high Ca^2+^ affinity biosensors Twitch-1 and Twitch-2B showed the highest ratio values in both the atrium and ventricle, whereas lower affinity TN-XXL and Twitch-4 exhibited lower ratio values. Figure 2B shows the average ratios over time of the representative embryos of Figure 2A in the atrium and ventricle. The traces of Twitch-4 were less noisy than those of the other biosensors. Thus, Twitch-4 required less smoothing than the other GECIs for the ratio analysis and extraction of the parameters (Table S3). In addition, Twitch-4 showed the largest relative ratio change between systole and diastole (ΔR/R_diastole_ = 4.8%) (Figure 2C and Table S1).

### 2.2. Ratiometric Imaging Reports Ca^2+^ Changes and Corrects for Motion Artifacts

**Figure 3:**
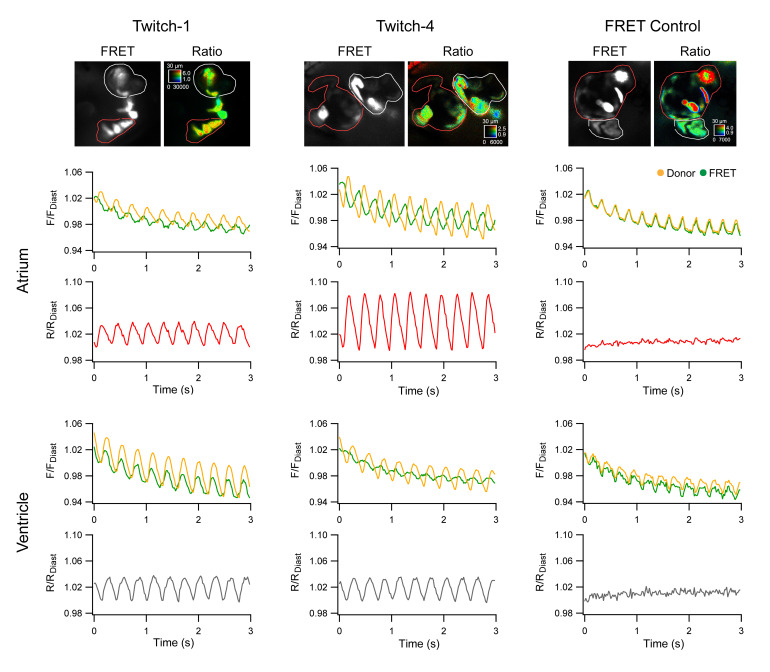
Ratiometric measurements with Twitch-1, Twitch-4, and a Ca^2+^-insensitive FRET control. The genetically encoded Ca^2+^ indicators (GECIs) Twitch-1 and Twitch-4, or a FRET construct insensitive to Ca^2+^ (ECFP-16aa-EYFP), were transiently expressed in the heart of zebrafish embryos. Regions-of-interest (ROI) were manually drawn on the atrium (red line) and ventricle (white line). The normalized change in fluorescence in the donor and FRET channels (F/F_diastole_) (upper graphs in atrium and ventricle) and ratio (R/R_diastole_) (lower graphs in atrium and ventricle) over time are shown. F_diastole_ and R_diastole_, used for normalization, were the fluorescence and ratio value of the first diastole. The embryos were treated with 75 µM PAB to decrease the contractions, but the hearts still retained some motion, which affected the intensity measurements. The FRET control showed that the emission ratio is largely indifferent to motion artifacts and corrected the photobleaching.

The motion during the cardiac cycle distinctly decreased by incubation with PAB, although in most embryos the heart still showed some movement (compare Supplementary Materials Videos S1 and S2). To verify that the change in emission ratio of the biosensors was reporting Ca^2+^ and was not an artifact caused by the remaining motion of the heart, we expressed a FRET construct with no Ca^2+^-binding domain, composed of ECFP and EYFP joined by a flexible linker of 16 amino acid residues [42]. Thus, in ECFP-16aa-EYFP, all the fluorescence changes were entirely due to motion. This construct, expressed under control of the *cmlc2* promoter, also labelled heart cells in a mosaic way (Figure 3, FRET Control). We applied the same imaging protocol as above, acquiring donor and FRET images (both with donor excitation). The intensity of the donor and FRET channels of the Ca^2+^-insensitive construct was indeed altered by motion of the heart and changed in parallel during beating, but this artifact was cancelled out in the ratio since the movement affected both emission channels equally (Figure 3; Video S5). In contrast, Twitch-1 and Twitch-4 showed clear oscillations in the ratio correlative with contractions in both the atrium and ventricle. The change in fluorescence intensity of Twitch-1 and Twitch-4 in the donor and FRET channels had a motion artifact component and a FRET change component, but the latter usually dominated over the former. Thus, the intensity of the donor and acceptor channels changed in opposite directions in most embryos, a hallmark of a change in FRET since the donor gets dim as energy is transferred to the acceptor. We concluded that the ratio of Twitch biosensors, by removing the influence of motion, only left the changes in the ratio that were Ca^2+^ dependent. Thus, the emission ratio changes observed with these GECIs indeed reflected changes in free Ca^2+^ concentration.

Incidentally, ratioing also corrected the photobleaching observed in the individual fluorescence channels of the Ca^2+^-insensitive control, Twitch-1 and Twitch-4 (Figure 3). Table S2 shows the percent photobleaching (~4.5%) of the various GECIs during 5 s of continuous illumination. Twitch-2B showed less photobleaching than the other biosensors (~2.5%) because it is composed of different FPs (Table 1) [24].

### 2.3. Basal Cardiac Ca^2+^ Kinetics Obtained with Each Biosensor

**Figure 4:**
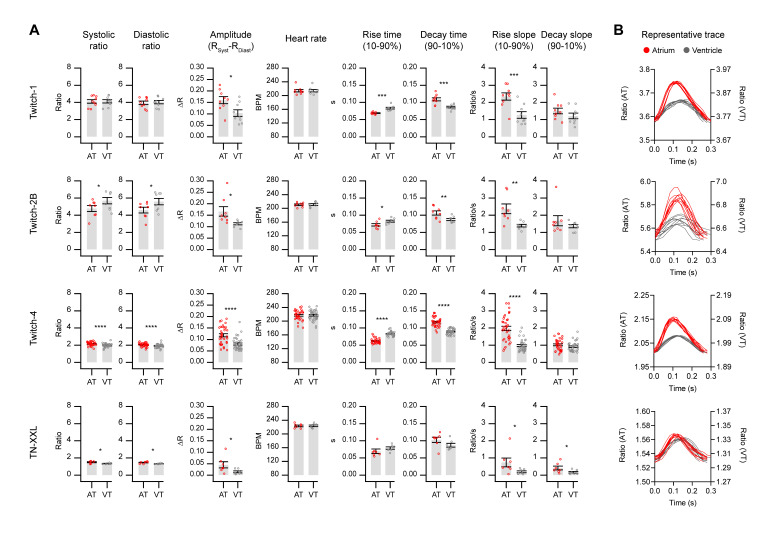
Basal cardiac Ca^2+^ kinetics obtained with each biosensor. (**A**) Several kinetic parameters of Ca^2+^ changes in the atrium (red) and the ventricle (gray) in 3 dpf zebrafish embryos were extracted with custom routines written in Igor Pro 8 software (see Methods and Figure 1 for definition of each parameter). The bars show the mean ± S.E.M and each dot represents one embryo, *n* = 7 to 35 embryos of one or four independent experiments. A paired Student’s *t*-test was used (* *p* < 0.05, ** *p* < 0.01, *** *p* < 0.001, **** *p* < 0.0001). (**B**) The shape of the Ca^2+^ transients in the atrium (red) and the ventricle (gray) during contractions of a representative embryo for each GECI is shown. The traces were aligned at the start of each peak in both the atrium and ventricle, thus the delay in ventricular versus atrial Ca^2+^ rise is not shown. Note the different ratio scales for the atrium (AT) and ventricle (VT).

In Figure 4 we show the parameters of the Ca^2+^ changes in the atrium and ventricle and the HR extracted from the ratio time courses in embryos expressing each biosensor, as was shown in Figure 1C. The systolic and diastolic ratio, the amplitude ΔR, and rise and decay slopes are factors that should vary between GECIs. In contrast, the parameters which depend only on time and should be independent of the probe are the HR, the rise time 10–90%, and the decay time 90–10% of the Ca^2+^ transients.

The emission ratios were between 4 and 5 for the high Ca^2+^ affinity biosensors Twitch-1 and Twitch-2B (0.25 and 0.2 µM *K*d, respectively), whereas the lower affinity TN-XXL and Twitch-4 (0.8 and 2.8 µM *K*d, respectively) showed ratios close to 1.4 and 2, respectively (Figure 4A and Table S5). Whereas Twitch-2B displayed higher ratios in the ventricle than in the atrium (*p* = 0.027 and 0.019 for systole and diastole, respectively), the opposite was true for Twitch-4 and TN-XXL. In Twitch-1, the ratios in the atrium and ventricle were similar. Because of these differences between the biosensors, we could not distinguish whether the Ca^2+^ levels were different in these chambers. We attempted an in situ calibration of the ratio in terms of absolute Ca^2+^ concentration with a Ca^2+^ ionophore. Although we observed changes in the ratios in the direction of saturation (with high extracellular Ca^2+^) and desaturation (with EGTA), we were unable to obtain reliable Rmin and Rmax values, probably because these compounds did not fully equilibrate the Ca^2+^ levels across the embryo skin and across the plasma membrane of cardiomyocytes. The use of a Ca^2+^-sensitive dye as a control does not seem feasible either. With intensiometric dyes (like fluo-4) the heart contraction results in motion artifacts. Furthermore, although labelling embryos might be possible with emission ratio dyes (like indo-1), we do not envision a way to restrict labelling to the embryo heart.

TN-XXL showed the smallest ΔR between systole and diastole, and Twitch-4 produced the largest relative ratio change (ΔR/R_diastole_) of all the biosensors (5.5% ± 1.7 in atrium; 4.1% ± 1.3 in ventricle). The ΔR was larger in the atrium than in the ventricle for all biosensors (Figure 4A and Table S5). While this may indicate that the increase in free Ca^2+^ in the atrium was larger than that of the ventricle, it could also have other causes. For instance, with the embryo orientation on the microscope stage used in this study, autofluorescence of the yolk overlapped somewhat with the atrium, but not with the ventricle, which could artifactually alter the ratio in the atrium. The autofluorescence peak was about 550 nm, overlapping with YFP emission, as was shown in wavelength scans taken by confocal microscopy (Figure S1A). Moreover, the fungicide methylene blue used in the E3 medium increased the yolk autofluorescence by approximately 5–10-fold and displaced the emission peak about 20 nm, as was shown in embryos maintained from Day 0 with or without methylene blue (Figure S1B).

The HR was identical in the atrium and ventricle, as expected. The average HR was 216.6 ± 12.6 bpm (*n* = 59 embryos with the four biosensors). Rise and decay times obtained with the various GECIs were similar (Table S4). The shape of the Ca^2+^ transients differed in the atrium and ventricle, and all GECIs showed qualitatively the same features (Figure 4B, representative traces). As described earlier with intensiometric biosensors [25,26,43], the upstroke of the Ca^2+^ transient was faster in the atrium than in the ventricle. The atrium showed a shorter rise time and longer decay time, whereas the Ca^2+^ transients in the ventricle were more symmetrical (rise and decay times were similar) (Figure 4A and Table S5). Likewise, the rise slope 10–90% (in ratio units/s) was larger in the atrium than in the ventricle for all biosensors. Zhang et al. [17] showed in patch-clamped cardiomyocytes from adult zebrafish that Ca^2+^ (measured with Fluo-4) did not reach a steady value at a stimulation frequency of 1 Hz during diastole, but it did at lower frequencies. Thus, there was an increase in diastolic Ca^2+^ levels at higher stimulation frequencies. At the relatively fast HR that we observed in 3 dpf embryos, neither the atrium nor the ventricle showed a stable Ca^2+^ baseline between heart beats (Figure 2 and Figure 3).

To sum up, the measurements in Figure 4 provided a detailed characterization of the Ca^2+^ dynamics in the embryo heart and showed that the biological findings were reproducible with the various probes, with some differences attributed to their dissimilar properties, such as Ca^2+^ affinity. These experiments provided the control values to be compared with the effects of drugs affecting heart function.

### 2.4. Influence of GECI Overexpression on Ca^2+^ Transients and on Functional Parameters of the Heart

We tested whether expression of GECI caused Ca^2+^ buffering in heart cells, possibly blunting the Ca^2+^ transients, or other deleterious effects. As Twitch-1 and Twitch-4 share the same fluorophores and were expressed under the same promoter, their concentrations in the heart cells would be expected to be similar in embryos injected with their respective cDNAs.

**Figure 5:**
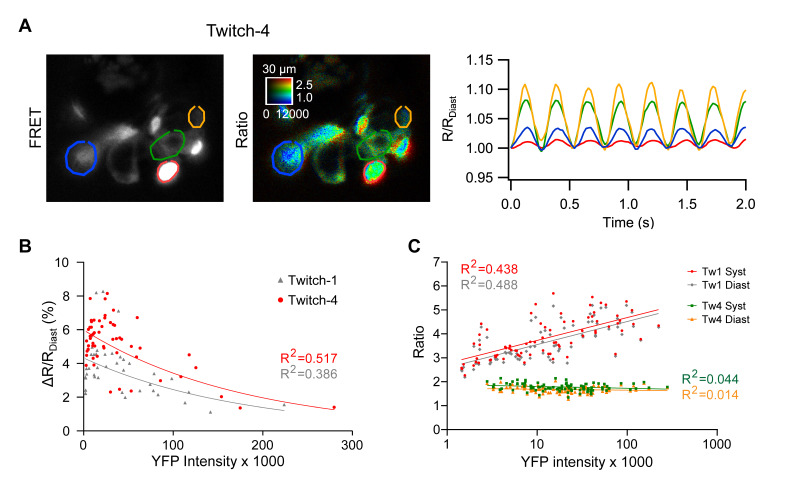
Effect of the expression level of the biosensors on the emission ratio and on the systolic ratio change (ΔR). (**A**) A representative experiment with variable expression of Twitch-4 between cells in the ventricle is shown (left, fluorescence intensity image of the FRET channel; right, ratio image in pseudo color). Regions-of-interest (ROI) were manually drawn on cells expressing increasing levels of biosensor (from yellow to green, blue and red). The color-coded traces in the graph correspond to the cells in the images. (**B**) Dependence of the relative ratio change (%[ΔR/R_diastole_]) on the expression level of Twitch-1 and Twitch-4 in the ventricle, estimated as the fluorescence intensity of directly excited YFP. Each dot represents one cell in 11 to 13 embryos of three independent experiments. Data were adjusted to an exponential model (for Twitch-1: r = −0.621, R^2^ = 0.386, *p* < 0.0001, *n* = 41; for Twitch-4: r = −0.719, R^2^ = 0.517, *p* < 0.0001, *n* = 56). (**C**) Effect of biosensor concentration (estimated by YFP fluorescence) on the emission ratio of Twitch-1 and Twitch-4, in systole and diastole (logarithmic scale). Data of Twitch-1 were adjusted to a logarithmic model (for R_systole_: r = 0.662, R^2^ = 0.438, *p* < 0.0001, *n* = 77; for R_diastole_: r = 0.699, R^2^ = 0.488, *p* < 0.0001, *n* = 77); the Twitch-4 ratio data did not correlate with YFP intensity.

The biosensor concentration differs between cells when they are expressed transiently, which can be a source of ratio variability and might explain the dispersion in ΔR observed in Figure 4. We deliberately chose embryos with large differences in expression levels between cells, as estimated by YFP fluorescence (excited directly). Cells with higher Twitch-4 expression levels exhibited lower ratio changes during systole than dimmer neighbors, particularly in the ventricle, where cells were more fluorescent than in the atrium (Figure 5A, representative experiment). The dot plots in Figure 5B show that there was an inverse relationship between the concentration of Twitch-1 and Twitch-4 in different cells and their relative ratio change during systole (% ΔR/R_diastole_): the higher the concentration of biosensor, the lower the systolic ratio change. This suggested blunted Ca^2+^ transients caused by buffering by the GECI (exogenous buffer) and/or less biosensor molecules exchanging Ca^2+^ ions in cells with high expression of the probes [23]. Other options can give rise to the same phenomenon. For example, overexpression could result in poorly folded proteins with Ca^2+^-insensitive fluorescence, or proteins that bind to intracellular components and lose sensitivity to Ca^2+^. In either case, that would result in a greater fluorescence background and less ΔR. It is difficult to distinguish between these two causes. If it were buffering one would expect the heart contraction and the kinetics of the oscillations to be altered and that did not seem to occur; if it were unfolded proteins, it should not.

In addition, the emission ratio of Twitch-1 increased with the expression level of the biosensor, an effect which was not observed in Twitch-4-expressing embryos (Figure 5C). This effect might be explained by intermolecular FRET in embryos expressing high concentration of Twitch-1, but it is nevertheless a puzzling observation, since the two GECIs are composed of the same FPs (Table 1) [24]. We consider that, in transgenic zebrafish expressing Twitch-1, the levels of the biosensor will be uniform between cells and this effect will be of little practical importance.

In conclusion, from the standpoint of the possible interference of GECI expression on the physiological Ca^2+^ transients, the lower the biosensor concentration compared to that of the endogenous buffers and the more homogeneous the level of expression, the better. However, a different aim would be to adjust the GECI expression levels to maximize the SNR in Ca^2+^ imaging studies. Rose et al. showed that the SNR of a biosensor is greatest at concentrations where the buffering capacities (*k*) of the exogenous and endogenous buffers are equal (*k_endogenous_* = *k_biosensor_*) [23]. In practice, a compromise must be reached between less interference with physiological Ca^2+^ signaling (Figure 5B) and optimization of the SNR. The biosensor concentration of TN-XXL in the heart of a transgenic mouse was found to be between 2.5 and 6 µM [34].

As mentioned in the Introduction, expression of biosensors composed of troponin C might potentially alter functional parameters such as the force of the cardiac contractions. To estimate the strength of the ventricular beats, we measured the ventricular fractional area change, equivalent to the clinically relevant ejection fraction. The HR and the fractional area change were estimated by transmitted light in beating hearts not treated with PAB. They did not change significantly between the control and Twitch-4-expressing embryos (Figure S2A). Thus, the expression of Twitch-4 in some ventricular myocytes (mosaic expression, Figure 2) did not affect the HR and cardiac contractility.

### 2.5. Effect of an L-Type Ca^2+^ Channel Blocker on Ca^2+^ Transients in the Zebrafish Heart

To determine whether these GECIs were useful to gauge the effect of drugs on heart Ca^2+^, we tested their response to well-known pharmacological agents acting on the heart, the LTCC blocker nifedipine and the β-adrenergic antagonist propranolol.

LTCCs are the major inward currents during the action potential plateau in human adult cardiomyocytes. Activators of these channels prolong the action potential duration and the QT interval of the electrocardiogram, whereas blockers have the opposite effect. In contrast to humans, various Ca^2+^ influx routes have been described in the zebrafish heart: LTCCs (Cav1.2 and 1.3 channels), T-type Ca^2+^ channels (TTCC, Cav3.1 and 3.2), and the NCX working in reverse mode [5,11,16,17].

**Figure 6:**
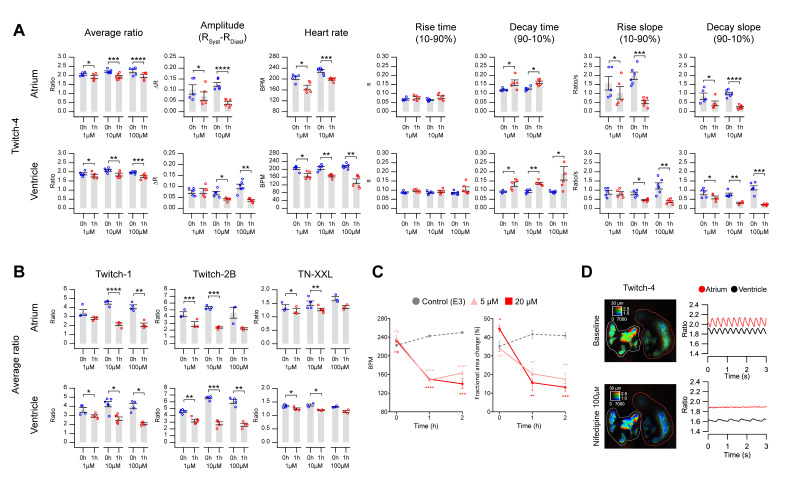
Effect of the L-type Ca^2+^ channel blocker nifedipine on heart Ca^2+^ levels measured with Twitch and TN-XXL biosensors. (**A**) Ca^2+^ kinetic parameters were measured in 3 dpf zebrafish embryos expressing Twitch-4 in the heart, before and after 1 h incubation with 1, 10, and 100 µM nifedipine. Different embryos were used for each nifedipine concentration. A paired Student’s *t*-test was used (* *p* < 0.05, ** *p* < 0.01, *** *p* < 0.001, **** *p* < 0.0001). (**B**) Average of the systolic and diastolic ratio values obtained in 3 dpf embryos expressing Twitch-1, Twitch-2, and TN-XXL, before and after 1 h incubation with 1, 10, and 100 µM nifedipine. Data are shown as the mean ± S.E.M., *n* = 3 to 6 embryos for each concentration, from one or two independent experiments. A paired Student’s *t*-test was used (* *p* < 0.05, ** *p* < 0.01, *** *p* < 0.001, **** *p* < 0.0001). (**C**) HR and fractional area change in uninjected embryos incubated with 5 and 20 µM nifedipine compared to control. These embryos were not incubated with PAB. Data are shown as the mean ± S.E.M., *n* = 3 to 4 embryos for each group, from one independent experiment. An unpaired Student’s *t*-test was used to compare each treatment with the control (* *p* < 0.05, ** *p* < 0.01, *** *p* < 0.001, **** *p* < 0.0001). (**D**) A representative embryo in which treatment with 100 µM nifedipine (bottom image and graph) decreased the Ca^2+^ levels and completely abrogated the contractions in the atrium. Regions-of-interest (ROI) were manually drawn on the atrium (red line) and ventricle (white line). The effect of nifedipine on the average ratio was more pronounced with the high affinity biosensors Twitch-1 and Twitch-2B than with the lower affinity Twitch-4 or TN-XXL, even at 1 and 10 µM nifedipine (Figure 6B and Table S7). At Ca^2+^ levels comparable to the *K*d, the fractions of Ca^2+^-bound and free biosensor are most sensitive to Ca^2+^. Thus, despite Twitch-4 generally providing a more robust reading of Ca^2+^ (larger ΔR/R_diastole_, less smoothing), in some cases the higher affinity GECIs may be advantageous.

We used the channel blocker nifedipine in Twitch-4-expressing embryos to determine the contribution of LTCC to Ca^2+^ changes in 3 dpf zebrafish embryos. As expected for a Ca^2+^ channel blocker, the Ca^2+^ levels decreased dose-dependently by incubation with nifedipine in both the atrium and ventricle (Figure 6A and Table S6, average ratio). The decrease in ΔR of the systolic Ca^2+^ rise was very significant, particularly in the atrium. Likewise, the HR decreased dose-dependently with nifedipine in both chambers. Atrial beating stopped altogether in embryos treated with 100 µM nifedipine (Figure 6D). Because of the lower HR, the decay time 90–10% of the Ca^2+^ transients increased in both chambers, but the rise time 10–90% did not change. Since ΔR decreased, the rise and decay slopes also decreased dose-dependently. It is worth noting that the concentrations of drugs indicated are those in the bath over the agarose layer embedding the embryos (Figure 1A). It takes time for drugs to diffuse through the agarose and several cell layers and thus the final concentration of the drug reaching the heart is not known. The solvent DMSO at the highest concentration used in his study (1%) did not change the kinetic parameters of the Ca^2+^ transients (Table S10), except for the ventricle rise slope.

Since nifedipine, even at low concentrations, decreased the Ca^2+^ levels, it should influence the force of contraction, which depends on the amount of Ca^2+^ bound to troponin. Nifedipine at 5 and 20 µM markedly decreased the HR and the ventricular fractional area change (Figure 6C and Table S8). Thus, in this model there was a functional effect of the inhibition of the Ca^2+^ transients by the channel blocker.

Some embryos stopped atrial beating altogether with 100 µM nifedipine and Ca^2+^ levels stopped oscillating. This provided an opportunity to estimate the SNR of the Ca^2+^ changes in the absence of drugs, taking as background noise the S.D. of the stable reading in the atrium after nifedipine for each biosensor (Figure 6D) (see Methods). Under these conditions, the SNR (ΔR/S.D._background_) of Twitch-1 was found to be 28.6 ± 8.3 in the atrium and 18.4 ± 7.7 in the ventricle (mean ± S.D.). That of Twitch-2B was 14.2 ± 5 in the atrium and 9.6 ± 1.2 in the ventricle. The SNR of Twitch-4 was 16.8 ± 5.5 in the atrium and 11.4 ± 3.9 in the ventricle, whereas for TN-XXL it was 16.9 ± 13.3 in the atrium and 5.7 ± 4.1 in the ventricle. Thus, Twitch-1 provided the best SNR of the studied GECIs, followed by Twitch-4.

### 2.6. Effect of a β-Adrenergic Antagonist on Ca^2+^ Transients in the Zebrafish Heart

Embryos at 3-5 dpf have been shown to respond to β adrenoreceptor ligands and demonstrate an adrenergic tone at 5 dpf, whereas the vagal (cholinergic) input matures later during development [25,44,45]. The stimulation of β1 adrenoreceptors has a positive chronotropic effect on the zebrafish heart whereas cholinergic receptors slow down the HR. Thus, the HR of 3 dpf embryos is about 210 bpm (this work) compared to 120–130 bpm in the adult [44].

**Figure 7:**
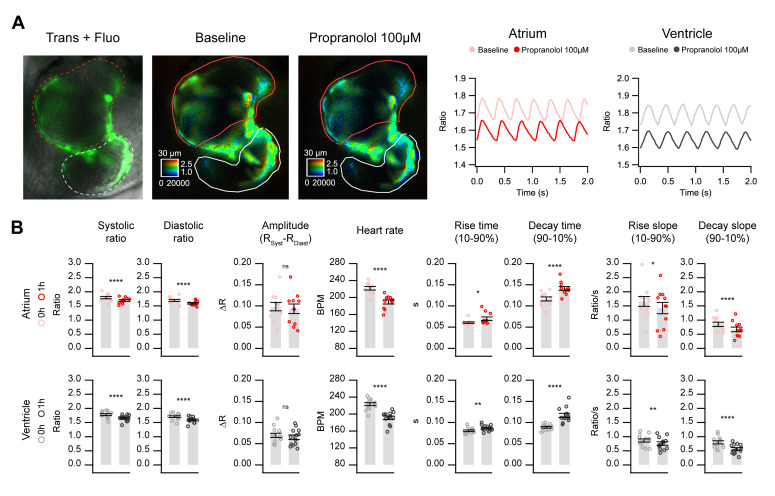
In vivo Ca^2+^ kinetics in the heart of Twitch-4-expressing embryos in response to propranolol. Imaging was performed before and after 1 h incubation with 100 µM propranolol in 3 dpf zebrafish embryos expressing Twitch-4 in the heart. (**A**) Overlay of transmitted light and fluorescence in a representative experiment (left image). The corresponding emission ratio images are shown in pseudo color before (middle) and after (right) 1 h incubation with propranolol. ROIs were drawn to delimit fluorescent cells in the atrium (red) and ventricle (white). The graphs show the oscillations of Ca^2+^ in the atrium and ventricle before and after propranolol. (**B**) Effect of propranolol on the kinetic parameters of the cardiac Ca^2+^ transients. Data are shown as the mean ± S.E.M., *n* = 11 and 13 embryos for atrium and ventricle, respectively, from two independent experiments. A paired Student’s *t*-test was used (* *p* < 0.05, ** *p* < 0.01, **** *p* < 0.0001, n.s. not significant).

We tested the effect of the β-adrenergic antagonist propranolol in embryos transiently expressing Twitch-4. Propranolol at 1 and 10 µM did not have a significant effect on any of the measured parameters. At 100 µM (1 h), it decreased the HR by 14% and decreased the systolic and diastolic Ca^2+^ levels in both chambers but did not affect the ΔR (Figure 7 and Table S9). It also increased the rise and decay time of the Ca^2+^ transients and decreased the rise and decay slopes in both the atrium and ventricle. To find out whether biosensor expression could alter the effects of a drug such as propranolol on the heart, the HR was measured in the control and Twitch-4-expressing embryos by transmitted light. The effect of propranolol (100 µM) on the HR was similar in the uninjected control embryos and in those expressing Twitch-4 (Figure S2B).

## 3. Discussion

In this work, we evaluated the usefulness of TN-XXL and Twitch GECIs to measure the Ca^2+^ levels in the heart of zebrafish embryos. The study revealed the benefits and drawbacks of each probe and served to assess which of them would be expected to perform better in a transgenic model for applications such as drug screening and pathophysiological studies of the heart.

We demonstrated that ratio measurements are well suited to correct for motion artifacts in a moving organ like the developing zebrafish heart, unlike single wavelength GECIs, which require complete blocking of the contractions, since the contribution of Ca^2+^ and motion to the fluorescence change cannot be separated from one another. In previous studies with intensiometric biosensors, contraction was prevented with morpholino oligomers targeted against *tnnt2a* (“silent heart” morpholino) or by using the myosin inhibitors 2,3-butanedione monoxime, blebbistatin, or PAB [6,25,43]. Although we used the non-fluorescent myosin inhibitor PAB to facilitate quantification in user-defined ROIs, heart contraction was not completely blocked (compare Videos S1 and S2). We ruled out that the observed changes in the emission ratio during contraction were caused by residual motion of the heart by using a FRET construct insensitive to Ca^2+^: while donor and FRET channel fluorescence were affected by movement, the fluorescence ratio was not (Figure 3). Thus, TN-XXL and Twitch GECIs reported fluctuating Ca^2+^ levels during the heart cycle, supporting the value of ratio imaging in moving specimens [23,27]. Compared to intensiometric GECIs, ratiometric biosensors provide the advantage of registering heart activity in a more physiological way. In a previous publication we observed that the Ca^2+^ changes during skeletal muscle contractions in zebrafish embryos expressing Twitch-4 were similar to those observed with the Ca^2+^ photoprotein GFP-aequorin [46]. In an earlier report, ratiometric voltage biosensors adequately corrected the movement artifacts in zebrafish [47].

The mosaicism caused by transient expression of the probes resulted in large differences in their concentration among cells. This allowed us to examine whether the biosensors, as exogenous buffers of Ca^2+^, interfered with the Ca^2+^ signals. When their buffering power exceeds that of the endogenous buffers, the Ca^2+^ biosensors may interfere with and slow down the physiological events triggered by the Ca^2+^ [23,29,30,31]. The ratio changes were inversely related to the biosensor brightness of a cell, taken as a proxy for the expression level. This could be caused by Ca^2+^ buffering in cells expressing high levels of GECIs for both low and high affinity probes, particularly in the ventricle (Figure 5). Alternatively, poorly folded proteins and/or Ca^2+^-insensitive biosensors as a result of overexpression could result in the same effect. Other potential deleterious off-target effects of biosensor expression were searched for. The mosaic expression of Twitch-4 in some cardiomyocytes did not affect the ventricular fractional area change during systole, an estimate of the force of contraction (Figure S2A). However, this will have to be re-evaluated in a stable expression model in which all cells express the biosensor. A gene profiling study in transgenic mice expressing TN-XXL demonstrated mild signatures of biosensor expression, but did not result in a regulation of endogenous Ca^2+^ buffers [34]. Notably, cardiomegaly was observed, an effect which should be investigated in transgenic zebrafish expressing Twitch biosensors.

We wrote routines in Igor Pro to automatically analyze the raw ratio values over time and extracted quantitative parameters to characterize the kinetics of the Ca^2+^ changes (Figure 4). The GECIs examined were able to detect changes in these parameters induced by an LTCC blocker and by a β-adrenergic antagonist (Figure 6 and Figure 7). As expected for an LTCC blocker, nifedipine decreased the HR, likely reflecting an effect on the pacemaker, as well as the amplitude of the systolic Ca^2+^ rise (ΔR). Since the cytoplasmic Ca^2+^ levels are tightly linked with the force of contraction, nifedipine decreased the fractional area change (Figure 6C).

In nifedipine-treated embryos, ΔR and the HR decreased much more in the atrium than in the ventricle (Figure 6A). Thus, 100 µM nifedipine caused arrest of the atrial Ca^2+^ changes and contraction, while the ventricle continued to beat at a slower pace (Figure 6D). Presumably it was excited from the sinoatrial node along the conduction system, or there could be a ventricular pace, since a pacemaker *If* current has been observed in the ventricle when the atrial action potentials were inhibited or there was an AV block [25,43]. The arrest of atrial contraction may be due to different expression or function of the LTCCs in the atrium and ventricle, or to the presence of additional Ca^2+^ influx pathways in the ventricle (like TTCCs and NCX in reverse mode). Thus, reverse mode NCX in adult zebrafish cardiomyocytes has been shown to contribute to Ca^2+^ influx at membrane potentials more positive than +10 mV [17]. Hou et al. showed that heart excitability matures differently in the atrium and ventricle [43]. The upstroke of the action potential in 54 hpf embryos depended mostly on LTCCs in both the atrium and ventricle, since action potentials were blocked by nifedipine but not by quinidine [43]. However, at 90–102 hpf, while the excitability of the atrium still depended largely on LTCCs, the excitability of the ventricle rested on Na^+^ channels (complete block by quinidine). Furthermore, Arnaout et al. [6] showed in 48 hpf embryos that the upstroke of the action potential in both the atrium and ventricle depended on LTCCs (it was sensitive to nifedipine, but not to tetrodotoxin), consistent with reports in early differentiated mammalian cardiomyocytes. Thus, there seems to be a transition from immature Ca^2+^-dependent excitation to a mature Na^+^-dependent one during heart development. In the zebrafish ventricle this transition occurs about 3 dpf and in the atrium about 4 dpf. The dependence of atrial excitation on LTCCs in 3 dpf embryos may therefore explain its sensitivity to nifedipine, compared to the ventricle.

We confirmed that 3 dpf zebrafish embryos have a strong sympathetic input to the heart or a state of activation of β1 adrenoreceptors, since the β-adrenergic blocker propranolol caused bradycardia and a decrease in the Ca^2+^ levels in both heart chambers (Figure 7). In agreement with these results, in 3 dpf transgenic embryos expressing GCaMP6f, propranolol showed a 26% reduction in the HR and a decrease in the diastolic Ca^2+^ levels [25].

There was a striking difference between the small ratio change during systolic Ca^2+^ transients (about 5% in Twitch-4) compared to the large ratio change of in vitro calibration (Table 1) [24]. Although the in vitro dynamic range of many modern biosensors is impressive, reaching several-fold between the zero and saturating Ca^2+^ concentrations, in cells, tissue slices, and in vivo, the fluorescence or ratio changes are much more modest during physiological responses. For instance, TN-XXL showed an in vitro ∆R/R of 260%, whereas the ratio increased only 1.6% during a single action potential in hippocampal slices [33]. In heart explants of a transgenic mouse expressing TN-XXL the ratio increased by 10% during systole [34]. Likewise, Twitch-2B showed an in vitro ∆R/R of 800%, whereas the ratio changed by 26.5% during an action potential in acute cortical slices [24]. Similarly, GCaMP6f fluorescence in vivo in the mouse visual cortex increased by only 19% during an action potential [19]. The ratio changes we observed during the cardiac cycle in embryo hearts with the TN-XXL and Twitch biosensors were in a similar range to those in these in vivo results.

The goal of the present study was to provide evidence to choose the best ratiometric biosensor(s) for constructing transgenic zebrafish lines. In conclusion, Twitch-4 stood out as providing the largest relative ΔR during the heart cycle, outperforming the other GECIs. As a result, smoothing of Twitch-4 raw data required a smaller number of points than the other biosensors (Table S3). This agrees with the reported in vitro calibration data, which showed a very linear response of Twitch-4 with Ca^2+^ (Hill slope of 1.04) and a 6-fold ratio difference between the Ca^2+^-free and Ca^2+^-saturated probe, the largest of these biosensors. Twitch-4 also has faster kinetics compared to the other GECIs (Table 1) [24]. However, the higher affinity Twitch-1 and Twitch-2b reported more sensitively the effects of nifedipine on Ca^2+^ levels; thus, they may be preferred to detect diastolic or decreased Ca^2+^ levels. In addition, Twitch-1 showed better SNR than Twitch-4. Twitch-2B might be problematic for use in the fast beating embryo heart as it has the slowest decay of these GECIs (time constant of 2.8 s) (Table 1), but it could still be useful to estimate the steady-state levels, for example, in models of heart failure. TN-XXL, which belongs to an earlier family of biosensors, showed the smallest ΔR and SNR of all.

The results on GECI overexpression suggested that the stable expression in a transgenic zebrafish line should be adjusted to provide sufficient SNR with the least possible deleterious effects of the added Ca^2+^ chelator [23]. In addition, stable or conditional expression should minimize the differences in the expression levels between cells observed here, improving the data quality of the experiments. A transgenic zebrafish line expressing a troponin-C-based ratiometric GECI could thus be invaluable to study Ca^2+^ heart physiology during development, to characterize the effects of arrhythmogenic or other drugs targeting the heart, and to detect the deleterious effects of drugs on heart function during pharmacological screening. Nevertheless, transient expression of these biosensors provides valuable insight into the physiology of Ca^2+^ changes during embryogenesis.

## 4. Materials and Methods

### 4.1. Constructs, Plasmids, and mRNA Synthesis

The Ca^2+^ biosensors Twitch-1, Twitch-2B, Twitch-4, and TN-XXL, a kind gift of Dr. Oliver Griesbeck (Max Planck Institute of Neurobiology, Martinsried, Germany), were cloned in the pDestTol2pA2 vector [36,37] under the cardiac-specific *cmlc2* promoter (*myl7*). Twitch-1, Twitch-4, and TN-XXL were cloned in the vector pTol2-*cmlc2*-MCS, designed to contain specific cloning sites and synthesized by a commercial supplier (GeneArt Gene Synthesis, Thermo Fisher Scientific, Waltham, MA, USA), at the *Eco*RI/*Bam*HI restriction sites. Twitch-2B and the Ca^2+^-insensitive FRET control ECFP-16aa-EYFP [42] were cloned in the vector pTol2(*cmlc2*:DsRed), a kind gift of Dr. S. Higashijima (Okazaki Institute for Integrative Bioscience, Okazaki, Japan), using the In-Fusion HD Cloning Plus kit (Takara Europe, Saint-Germain-en-Laye, France). pTol2(*cmlc2*:DsRed) was digested at the *Bam*HI/*Xba*I restriction sites to replace DsRed with Twitch-2B or ECFP-16aa-YFP. All constructs were confirmed by DNA sequencing (Stab Vida, Caparica, Portugal). Transposase mRNA was synthesized in vitro from vector pCS-zT2TP [36,37] using the mMESSAGE mMACHINE SP6 kit (Ambion Inc., Austin, TX, USA) following the manufacturer’s protocol.

### 4.2. Collection and Maintenance of Zebrafish Embryos

Wild-type AB zebrafish (*Danio rerio*) were kept in the Center for Animal Experimentation of the Albacete School of Medicine with a light/dark cycle of 14/10 h. Fertilized zebrafish eggs in the synchronized stage were obtained following standard procedures and maintained in E3 medium (5 mM NaCl, 0.17 mM KCl, 0.33 mM MgSO_4_, 0.33 mM CaCl_2_, 0.002% methylene blue, pH 7.4 in double distilled water) at 28.5 °C. All animal procedures were carried out in compliance with the UCLM Animal Experimentation Ethics Committee following national and EU regulations (approval identification code 900823 dated 30 May 2016, Consejería de Agricultura, Medio Ambiente y Desarrollo Rural, JCCM, Spain). All the experiments were conducted on zebrafish embryos and larvae of less than 120 hpf.

### 4.3. Microinjection of Eggs

Transient expression of the biosensors was obtained by the co-injection of transposase mRNA and the cDNA of each biosensor. Microinjection needles were prepared with glass capillaries of 1 mm external diameter (TW 100-F, World Precision Instruments, Sarasota, FL, USA) in a horizontal puller (Model P-97, Sutter Instrument Co., Novato, CA, USA) at 589 °C. Microinjection was performed manually on the blastodisc in 1 or 2 cell stage fertilized eggs through the vegetal pole using a microinjection unit (Femtojet, Eppendorf, Hamburg, Germany). The injection volume was 1–5 nL and the amount of mRNA and cDNA was ~150 and ~250 pg/egg, respectively. The injection solutions were diluted in 0.5% phenol red as an indicator of the injection volume. The injected eggs were kept in an incubator at 28.5 °C for optimum development up to 3 dpf. The staging of the embryos was carried out as described [48].

### 4.4. Mounting of Embryos for Microscopy

In order to reduce movement artifacts during imaging, the myosin inhibitor para-amino blebbistatin (PAB) (Optopharma, Budapest, Hungary) [40] was used to uncouple cardiac excitation and the increase in Ca^2+^ levels during systole from contraction. Embryos of 72 hpf were treated with 75 µM PAB for 2 h before mounting for microscopy. The embryos were embedded in 100 µL of 0.3% low melting point agarose in E3 medium containing 75 µM PAB, preheated to 42 °C and gelled on 96-well plates with square wells and a flat clear bottom (ibidi, Gräfelfing, Germany). Once solidified, 100 µL of E3 medium at 28 °C was added. For the transmitted light experiments, the embryos were not treated with PAB and were embedded in low melting point agarose without PAB. After mounting, the embryos were incubated for 30 min at 28 °C to allow for their recovery. For both widefield transmitted light and fluorescence experiments, the embryos were not anesthetized.

### 4.5. Confocal Microscopy

Embryos were maintained from Day 0 with or without methylene blue as indicated. Agar-embedded 3 dpf embryos were imaged in an inverted Axio Observer LSM710 confocal microscope (Carl Zeiss, Oberkochen, Germany) to obtain spectral image stacks (x, y, λ). A PlanApo 10x/0.45 NA objective and Zen2.3 software (Carl Zeiss, Oberkochen, Germany) were used. The acquisition was configured to use the Lambda scanning mode with laser excitation at 458 nm and a 464–718 nm emission window. In this mode, images were scanned in x–y, and the emission from each point is spectrally dispersed so the entire spectrum is simultaneously gathered by a detection array with up to 32 channels of 9.8 nm bandwidth each. The average intensity in ROIs drawn on the image λ stack was plotted along the wavelength axis to obtain the emission spectra.

### 4.6. Ratiometric Fluorescence Imaging of TN-XXL, Twitch-1, Twitch-2b and Twitch-4

We acquired fluorescence images of the heart in 72 hpf embryos expressing the ratiometric fluorescent biosensors TN-XXL, Twitch-1, Twitch-2b and Twitch-4 with a wide-field fluorescence microscope (DMIRE-2, Leica Microsystems, Wetzlar, Germany) equipped with a sCMOS camera (2048 × 2048 pixels, ORCA-Flash 4.0, Hamamatsu Photonics, Hamamatsu, Japan), controlled by the software Aquacosmos 2.6 (Hamamatsu Photonics, Hamamatsu, Japan). The image acquisition rate was 50 Hz (20 ms integration per image) during 5–10 s; some embryos were imaged at 100 Hz. To image at this speed, the donor (ECFP or mCerulean3) and acceptor (cpCitrine174 or cpVenusCD) FRET images were acquired simultaneously with an image splitter (W-View Gemini, Hamamatsu Photonics, Hamamatsu, Japan), which divided the camera field in two halves corresponding to donor and acceptor emission. During imaging the embryos were kept in a chamber incubator (PeCon GmbH, Erbach, Germany) at 28 °C. We define an experiment as the group of embryos from the same zebrafish crossing imaged in one day.

The embryos were excited continuously for 5–10 s with a LED source (Lambda TLED+, Sutter Instrument, Novato, CA, USA) using a 440AF21 nm bandpass filter (Chroma, Bellows Falls, VT, USA) and a beamsplitter (455DLRP, Omega Optical, Brattleboro, VT, USA). A 10x air objective (HC PlanApo 0.45 NA, Leica Microsystems, Wetzlar, Germany) was used. Fluorescence passed through the image splitter equipped with emission filters 483/32 nm and 542/27 nm (Semrock, Rochester, NY, USA), separated with a beamsplitter 509-FDi01 (Semrock, Rochester, NY, USA). With this configuration, the resolution of the images was 0.725 µm × 0.725 µm/pixel and the total field of view after image splitting was 742.4 µm (H) × 1484.8 µm (V). Transmission images were acquired to correlate the fluorescence with the anatomical structures of the embryo.

The FRET image corresponds to the cpCitrine174, cpVenusCD, or EYFP emission (542/27 nm) at the donor excitation (440AF21 nm), and the donor image corresponds to the ECFP or mCerulean3 emission (483/32 nm) at the donor excitation (440AF21 nm).

For quantification of the expression level of the biosensors, YFP was directly excited using cube HC-YFP (Semrock, Rochester, NY, USA), composed of an exciter at 500/24 nm, beamsplitter 520LP, and emission filter 542/27 nm. The filters within the emission image splitter (Gemini, Hamamatsu Photonics, Hamamatsu, Japan) were as indicated above, but only the YFP emission channel was used.

For drug response experiments, 10 mM stocks of nifedipine and propranolol (Sigma-Aldrich, Darmstadt, Germany) were made in dimethyl sulfoxide (DMSO). The stocks were diluted to 3, 30, and 300 µM in E3 medium at 28 °C. After recording the baseline images, 100 µL of E3 medium, propranolol, or nifedipine solution was added to the wells, reaching a final drug concentration of 1, 10, and 100 µM. Embryos were incubated for 1 h and a second set of images was taken.

### 4.7. Image Processing and Data Analysis

Ratio images and ratio data were analyzed with custom routines written in IGOR Pro 8 (WaveMetrics, Lake Oswego, OR, USA). The ratio FRET image/donor image was calculated pixel-by-pixel for each data point in time. Correction for image shift was done prior to the ratio calculation. The ratio value for an ROI was calculated by averaging all the individual pixels in that ROI, weighted by their intensity [28,39]. The Savitzky–Golay algorithm was applied to smooth the raw ratio changes with various numbers of points, depending on the noise of the recordings (Table S3). Several kinetic parameters of the Ca^2+^ transients were automatically calculated from the smoothed ratio data with a custom written routine in Igor Pro 8 (WaveMetrics, Lake Oswego, OR, USA): diastolic ratio (the lowest ratio in the cardiac cycle), systolic ratio (the highest ratio in the cardiac cycle), ΔR (systolic minus diastolic ratio), HR (in bpm), rise time (time from 10 to 90% of systolic Ca^2+^ rise), decay time (time from 90 to 10% of diastolic Ca^2+^ decay), rise slope (ΔR divided by the rise time, both values taken from 10 to 90% of the rise phase) and decay slope (ΔR divided by the decay time, both values taken from 90 to 10% of the decay phase). Data shown for each embryo represent the average of the cardiac cycles in 5 or 10 s of continuous recording (typically about 18 or 36 cardiac cycles, respectively), as indicated.

Transmitted light images were analyzed manually in ImageJ [49]. For the calculation of the HR by transmitted light, one ROI was placed just outside the edge of the ventricle during systole. The intensity changed whenever a diastole occurred because of relaxation of the heart wall; the HR was taken as the number of peaks per minute. To evaluate the contractility, the ventricular fractional area change was calculated from frames at end-diastole and end-systole (fully dilated and fully contracted ventricle, respectively) [50]. The area of the external edge of the ventricle was measured because the internal edge showed less contrast. The ventricular fractional area change was the difference between the end-diastolic and end-systolic areas, divided by the end-diastolic area, and was expressed as a percentage.

We calculated the SNR of the biosensors as the systolic ΔR divided by the S.D. of the background ratio (SNR = ΔR/S.D._background_). As the Ca^2+^ levels did not reach a steady value during diastole, we used as background signal the stable ratio obtained after treatment with 100 µM nifedipine in embryos in which the Ca^2+^ changes in the atrium stopped altogether (Figure 6D). The average of the SNR of 9, 8, 35, and 7 embryos for Twitch-1, Twitch-2B, Twitch-4, and TN-XXL, respectively, was calculated in the atrium and ventricle.

### 4.8. Statistical Analysis

Statistical analysis was done with Igor Pro 8 (WaveMetrics, Lake Oswego, OR, USA) and GraphPad Prism 6 (Graphpad Software, San Diego, CA, USA). All data in the figures are shown as the mean ± standard error of the mean (S.E.M.). The statistical significance between two groups was determined using a paired or unpaired Student’s *t*-test, as indicated. The correlation between the ratio change and fluorescence intensity was analyzed using a linear regression analysis of the transformed data with SPSS (IBM, Armonk, NY, USA). A *p* < 0.05 was considered statistically significant (* *p* < 0.05, ** *p* < 0.01, *** *p* < 0.001, **** *p* < 0.0001).

## Figures and Tables

**Table 1:** In vitro properties of the selected biosensors.

Biosensor	FRET Pair	Troponin C	Linkers	ΔR/R In Vitro (%)	ΔR/R Ex Vivo (%)	*K*d (µM)	Decay Time (s)	Hill Slope
Twitch-1	ECFP cpCit174	*Opsanus tau* swim bladder and white muscle	P, P	400		0.25	0.8	1.18
Twitch-2B	mCerulean3 cpVenusCD	*Opsanus tau* swim bladder and white muscle	VADA, PIYP	800	26.5 ^†^	0.2	2.8	1.31
Twitch-4	ECFP cpCit174	*Opsanus tau* swim bladder and white muscle	DA, PIY	600		2.8	0.5	1.04
TN-XXL *	ECFP cpCit174	Chicken skeletal muscle	RML, EL	260	10 ^‡^	0.8	0.88	1.5

* TN-XXL data from [33]. The *K*d was obtained in vitro and the decay time in a preparation of neuromuscular junction of *Drosophila melanogaster*. ^†^ Twitch-2B during a single action potential in mouse acute cortical slices [24]. ^‡^ TN-XXL during systole in heart explants from a transgenic mouse [34].

## Supplementary Materials

Supplementary Materials can be found at https://www.mdpi.com/1422-0067/21/18/6610/s1.

## Abbreviations

bpm	beats per minute
cpCitrine174	citrine fluorescent protein circularly permuted at amino acid 174
DMSO	dimethyl sulfoxide
dpf	days post-fertilization
ECFP	enhanced cyan fluorescent protein
FP	fluorescent protein
FRET	Fluorescence/Förster resonance energy transfer
GECI	genetically-encoded Ca^2+^ indicator
GFP	green fluorescent protein
hpf	hours post-fertilization
HR	heart rate
LTCC	L-type Ca^2+^ channel
NCX	Na-Ca exchanger
PAB	para-amino blebbistatin
ROI	region of interest
SNR	signal-to-noise ratio
TTCC	T-type Ca^2+^ channel

## References

[1] Brunello L., Slabaugh J.L., Radwanski P.B., Ho H.T., Belevych A.E., Lou Q., Chen H., Napolitano C., Lodola F., Priori S.G. (2013). Decreased RyR2 refractoriness determines myocardial synchronization of aberrant Ca^2+^ release in a genetic model of arrhythmia. Proc. Natl. Acad. Sci. USA.

[2] Landstrom A.P., Dobrev D., Wehrens X.H.T. (2017). Calcium signaling and cardiac arrhythmias. Circ. Res..

[3] Nemec J., Kim J.J., Salama G. (2016). The link between abnormal calcium handling and electrical instability in acquired long QT syndrome—Does calcium precipitate arrhythmic storms?. Prog. Biophys. Mol. Biol..

[4] Macrae C.A. (2010). Cardiac Arrhythmia: In vivo screening in the zebrafish to overcome complexity in drug discovery. Expert Opin. Drug Discov..

[5] Alday A., Alonso H., Gallego M., Urrutia J., Letamendia A., Callol C., Casis O. (2014). Ionic channels underlying the ventricular action potential in zebrafish embryo. Pharmacol. Res..

[6] Arnaout R., Ferrer T., Huisken J., Spitzer K., Stainier D.Y., Tristani-Firouzi M., Chi N.C. (2007). Zebrafish model for human long QT syndrome. Proc. Natl. Acad. Sci. USA.

[7] Haverinen J., Hassinen M., Dash S.N., Vornanen M. (2018). Expression of calcium channel transcripts in the zebrafish heart: Dominance of T-type channels. J. Exp. Biol..

[8] Hodgson P., Ireland J., Grunow B. (2018). Fish, the better model in human heart research? Zebrafish Heart aggregates as a 3D spontaneously cardiomyogenic in vitro model system. Prog. Biophys. Mol. Biol..

[9] Milan D.J., Jones I.L., Ellinor P.T., MacRae C.A. (2006). In vivo recording of adult zebrafish electrocardiogram and assessment of drug-induced QT prolongation. Am. J. Physiol. Heart Circ. Physiol..

[10] Nemtsas P., Wettwer E., Christ T., Weidinger G., Ravens U. (2010). Adult zebrafish heart as a model for human heart? An electrophysiological study. J. Mol. Cell. Cardiol..

[11] Van Opbergen C.J.M., van der Voorn S.M., Vos M.A., de Boer T.P., van Veen T.A.B. (2018). Cardiac Ca(2+) signalling in zebrafish: Translation of findings to man. Prog. Biophys. Mol. Biol..

[12] Brown D.R., Samsa L.A., Qian L., Liu J. (2016). Advances in the Study of Heart Development and Disease Using Zebrafish. J. Cardiovasc. Dev. Dis..

[13] Burns C.G., Milan D.J., Grande E.J., Rottbauer W., MacRae C.A., Fishman M.C. (2005). High-throughput assay for small molecules that modulate zebrafish embryonic heart rate. Nat. Chem. Biol..

[14] Cornet C., Calzolari S., Minana-Prieto R., Dyballa S., van Doornmalen E., Rutjes H., Savy T., D’Amico D., Terriente J. (2017). ZeGlobalTox: An Innovative Approach to Address Organ Drug Toxicity Using Zebrafish. Int. J. Mol. Sci..

[15] Letamendia A., Quevedo C., Ibarbia I., Virto J.M., Holgado O., Diez M., Izpisua Belmonte J.C., Callol-Massot C. (2012). Development and validation of an automated high-throughput system for zebrafish in vivo screenings. PLoS ONE.

[16] Bovo E., Dvornikov A.V., Mazurek S.R., de Tombe P.P., Zima A.V. (2013). Mechanisms of Ca(2)+ handling in zebrafish ventricular myocytes. Pflug. Arch. Eur. J. Physiol..

[17] Zhang P.C., Llach A., Sheng X.Y., Hove-Madsen L., Tibbits G.F. (2011). Calcium handling in zebrafish ventricular myocytes. Am. J. Physiol. Regul. Integr. Comp. Physiol..

[18] Akerboom J., Chen T.W., Wardill T.J., Tian L., Marvin J.S., Mutlu S., Calderon N.C., Esposti F., Borghuis B.G., Sun X.R. (2012). Optimization of a GCaMP calcium indicator for neural activity imaging. J. Neurosci. Off. J. Soc. Neurosci..

[19] Chen T.W., Wardill T.J., Sun Y., Pulver S.R., Renninger S.L., Baohan A., Schreiter E.R., Kerr R.A., Orger M.B., Jayaraman V. (2013). Ultrasensitive fluorescent proteins for imaging neuronal activity. Nature.

[20] Nakai J., Ohkura M., Imoto K. (2001). A high signal-to-noise Ca^2+^ probe composed of a single green fluorescent protein. Nat. Biotechnol..

[21] Tian L., Hires S.A., Mao T., Huber D., Chiappe M.E., Chalasani S.H., Petreanu L., Akerboom J., McKinney S.A., Schreiter E.R. (2009). Imaging neural activity in worms, flies and mice with improved GCaMP calcium indicators. Nat. Methods.

[22] Miyawaki A., Llopis J., Heim R., McCaffery J.M., Adams J.A., Ikura M., Tsien R.Y. (1997). Fluorescent indicators for Ca^2+^ based on green fluorescent proteins and calmodulin. Nature.

[23] Rose T., Goltstein P.M., Portugues R., Griesbeck O. (2014). Putting a finishing touch on GECIs. Front. Mol. Neurosci..

[24] Thestrup T., Litzlbauer J., Bartholomaus I., Mues M., Russo L., Dana H., Kovalchuk Y., Liang Y., Kalamakis G., Laukat Y. (2014). Optimized ratiometric calcium sensors for functional in vivo imaging of neurons and T lymphocytes. Nat. Methods.

[25] Van Opbergen C.J.M., Koopman C.D., Kok B.J.M., Knopfel T., Renninger S.L., Orger M.B., Vos M.A., van Veen T.A.B., Bakkers J., de Boer T.P. (2018). Optogenetic sensors in the zebrafish heart: A novel in vivo electrophysiological tool to study cardiac arrhythmogenesis. Theranostics.

[26] Weber M., Scherf N., Meyer A.M., Panakova D., Kohl P., Huisken J. (2017). Cell-accurate optical mapping across the entire developing heart. eLife.

[27] Koopman C.D., Zimmermann W.H., Knopfel T., de Boer T.P. (2017). Cardiac optogenetics: Using light to monitor cardiac physiology. Basic Res. Cardiol..

[28] Tsien R.Y., Harootunian A.T. (1990). Practical design criteria for a dynamic ratio imaging system. Cell Calcium.

[29] Tsien R.Y., Klee E.C.C. (1999). Monitoring cell calcium. Calcium as a Cellular Regulator.

[30] McMahon S.M., Jackson M.B. (2018). An Inconvenient Truth: Calcium Sensors Are Calcium Buffers. Trends Neurosci..

[31] Neher E., Augustine G.J. (1992). Calcium gradients and buffers in bovine chromaffin cells. J. Physiol..

[32] Eisner D.A., Caldwell J.L., Kistamas K., Trafford A.W. (2017). Calcium and Excitation-Contraction Coupling in the Heart. Circ. Res..

[33] Mank M., Santos A.F., Direnberger S., Mrsic-Flogel T.D., Hofer S.B., Stein V., Hendel T., Reiff D.F., Levelt C., Borst A. (2008). A genetically encoded calcium indicator for chronic in vivo two-photon imaging. Nat. Methods.

[34] Direnberger S., Mues M., Micale V., Wotjak C.T., Dietzel S., Schubert M., Scharr A., Hassan S., Wahl-Schott C., Biel M. (2012). Biocompatibility of a genetically encoded calcium indicator in a transgenic mouse model. Nat. Commun..

[35] Heim N., Griesbeck O. (2004). Genetically encoded indicators of cellular calcium dynamics based on troponin C and green fluorescent protein. J. Biol. Chem..

[36] Kawakami K., Takeda H., Kawakami N., Kobayashi M., Matsuda N., Mishina M. (2004). A transposon-mediated gene trap approach identifies developmentally regulated genes in zebrafish. Dev. Cell.

[37] Urasaki A., Morvan G., Kawakami K. (2006). Functional dissection of the Tol2 transposable element identified the minimal cis-sequence and a highly repetitive sequence in the subterminal region essential for transposition. Genetics.

[38] Huang C.J., Tu C.T., Hsiao C.D., Hsieh F.J., Tsai H.J. (2003). Germ-line transmission of a myocardium-specific GFP transgene reveals critical regulatory elements in the cardiac myosin light chain 2 promoter of zebrafish. Dev. Dyn. Off. Publ. Am. Assoc. Anat..

[39] Polito M., Vincent P., Guiot E. (2014). Biosensor imaging in brain slice preparations. Methods Mol. Biol..

[40] Varkuti B.H., Kepiro M., Horvath I.A., Vegner L., Rati S., Zsigmond A., Hegyi G., Lenkei Z., Varga M., Malnasi-Csizmadia A. (2016). A highly soluble, non-phototoxic, non-fluorescent blebbistatin derivative. Sci. Rep..

[41] Bakkers J. (2011). Zebrafish as a model to study cardiac development and human cardiac disease. Cardiovasc. Res..

[42] Domingo B., Sabariegos R., Picazo F., Llopis J. (2007). Imaging FRET standards by steady-state fluorescence and lifetime methods. Microsc. Res. Tech..

[43] Hou J.H., Kralj J.M., Douglass A.D., Engert F., Cohen A.E. (2014). Simultaneous mapping of membrane voltage and calcium in zebrafish heart in vivo reveals chamber-specific developmental transitions in ionic currents. Front. Physiol..

[44] Schwerte T., Prem C., Mairosl A., Pelster B. (2006). Development of the sympatho-vagal balance in the cardiovascular system in zebrafish (Danio rerio) characterized by power spectrum and classical signal analysis. J. Exp. Biol..

[45] Steele S.L., Yang X., Debiais-Thibaud M., Schwerte T., Pelster B., Ekker M., Tiberi M., Perry S.F. (2011). In vivo and in vitro assessment of cardiac beta-adrenergic receptors in larval zebrafish (Danio rerio). J. Exp. Biol..

[46] Vicente M., Salgado-Almario J., Soriano J., Burgos M., Domingo B., Llopis J. (2019). Visualization of Mitochondrial Ca^2+^ Signals in Skeletal Muscle of Zebrafish Embryos with Bioluminescent Indicators. Int. J. Mol. Sci..

[47] Tsutsui H., Higashijima S., Miyawaki A., Okamura Y. (2010). Visualizing voltage dynamics in zebrafish heart. J. Physiol..

[48] Kimmel C.B., Ballard W.W., Kimmel S.R., Ullmann B., Schilling T.F. (1995). Stages of embryonic development of the zebrafish. Dev. Dyn. Off. Publ. Am. Assoc. Anat..

[49] Schneider C.A., Rasband W.S., Eliceiri K.W. (2012). NIH Image to ImageJ: 25 Years of image analysis. Nat. Methods.

[50] Haendchen R.V., Wyatt H.L., Maurer G., Zwehl W., Bear M., Meerbaum S., Corday E. (1983). Quantitation of regional cardiac function by two-dimensional echocardiography. I. Patterns of contraction in the normal left ventricle. Circulation.

