# Supplementary material for: Mapping Calcium Dynamics in the Heart of Zebrafish Embryos with Ratiometric Genetically Encoded Calcium Indicators
Source: Int J Mol Sci. 2020 Sep 10;21(18):6610. doi: 10.3390/ijms21186610 (PMC7555812; doi:10.3390/ijms21186610)
Supplement: Supplementary file 1 [file ijms-21-06610-s001.zip › Supplementary Material Legend.pdf]

International Journal of *Molecular Sciences*

MDPI

Article

Mapping Calcium Dynamics in the Heart of Zebrafish Embryos with Ratiometric Genetically Encoded Calcium Indicators

Jussep Salgado-Almario [1], Manuel Vicente [1], Pierre Vincent [2,*], Beatriz Domingo [1,*] and Juan Llopis [1,*]

[1] Physiology and Cell Dynamics, Centro Regional de Investigaciones Biomédicas (CRIB), Facultad de Medicina de Albacete, Departamento de Ciencias Médicas, Universidad de Castilla-La Mancha, C/Almansa 14, 02006 Albacete, Spain; Jussep.Salgado@uclm.es (J.S.-A.); Manuel.Vicente@uclm.es (M.V.)
[2] UMR8256, Biological Adaptation and Ageing, CNRS, Sorbonne Université, F-75005 Paris, France
* Correspondence: pierre.vincent@sorbonne-universite.fr (P.V.); beatriz.domingo@uclm.es (B.D.); juan.llopis@uclm.es (J.L.)

Supplementary Materials

1. SUPPLEMENTARY TABLES

Table S1. Data for Figure 2C. In vivo ratiometric imaging of zebrafish heart Ca^{2+} dynamics with TnC-based biosensors.

Biosensor	$\Delta R/R_{diastole}$ (%)		
	n	Average	SD
Twitch-1	18	3.19	1.21
Twitch-2B	16	2.80	1.10
Twitch-4	70	4.79	1.64
TN-XXL	14	2.00	1.82

Table S2. Percent photobleaching (% $\Delta F/F_0$) observed in donor and FRET channels after 5 s of continuous illumination in the atrium and ventricle.

Biosensor	*n*	Atrium		Ventricle	
		Donor	FRET	Donor	FRET
Twitch-1	5	4.55 ± 0.97	4.5 ± 1.10	4.61 ± 1.18	4.79 ± 1.36
Twitch-2B	5	2.70 ± 0.50	3.01 ± 0.58	2.48 ± 1.30	3.63 ± 1.15

Twitch-4	5	4.01 ± 0.43	4.54 ± 0.85	4.28 ± 0.94	4.26 ± 0.61
TN-XXL	5	4.49 ± 0.59	4.36 ± 0.46	4.48 ± 0.49	4.31 ± 0.41

Table S3. Smoothing points required to analyze the biosensors with the Savitzky-Golay algorithm.

**Biosensor**	**Smoothing points**		
	n	**Average**	**SD**
Twitch-1	9	8.3	2
Twitch-2B	8	8.5	1.4
Twitch-4	35	5.8	1.5
TN-XXL	7	8.4	3.2

Table S4. Rise and decay time of the Ca^{2+} transients are independent of biosensor.

**Parameter**	**Biosensor**	**Atrium**				**Ventricle**			
		n	**Average**	**SD**	***p*-value (one-way ANOVA)**	***n***	**Average**	**SD**	***p*-value (one-way ANOVA)**
Rise time (s)	Twitch-1	9	0.069	0.003	***0.0099***	9	0.083	0.007	0.54
	Twitch-2B	8	0.072	0.01		8	0.083	0.008	
	Twitch-4	35	0.062	0.007		35	0.083	0.006	
	TN-XXL	7	0.070	0.017		7	0.079	0.009	
Decay time (s)	Twitch-1	9	0.109	0.014	0.067	9	0.087	0.008	0.80
	Twitch-2B	8	0.107	0.018		8	0.088	0.009	
	Twitch-4	35	0.117	0.013		35	0.089	0.008	
	TN-XXL	7	0.103	0.02		7	0.087	0.014	

**Atrium**		
**Parameter**	**Multiple comparisons test (Tukey Test)**	***p*-value**
Rise time (s)	Twitch-2B vs Twitch-4	***0.028***
	Twitch-2B vs Twitch-1	0.883

Twitch-2B vs TN-XXL	0.953
TN-XXL vs Twitch-4	0.176
TN-XXL vs Twitch-1	0.998
Twitch-1 vs Twitch-4	0.178

Table S5. Data for Figure 4A. Basal cardiac Ca^{2+} kinetics obtained with each biosensor.

**Parameter**	**Twitch-1**							**Twitch-2B**						
	**Atrium**			**Ventricle**			**p-value (*t*-test)**	**Atrium**			**Ventricle**			**p-value (*t*-test)**
	n	**Average**	**SD**	***n***	**Average**	**SD**		***n***	**Average**	**SD**	***n***	**Average**	**SD**	
Systolic ratio	9	4.12	0.63	9	4.13	0.59	0.901	8	4.77	0.95	8	5.70	1.06	***0.027***
Diastolic ratio	9	3.96	0.61	9	4.03	0.57	0.578	8	4.60	0.93	8	5.59	1.05	***0.019***
Amplitude	9	0.16	0.047	9	0.10	0.04	***0.025***	8	0.17	0.058	8	0.11	0.013	***0.027***
Heart rate (BPM)	9	214.0	10.35	9	214.1	9.82	0.80	8	210.2	6.4	8	211.1	7.3	0.11
Rise time (s)	9	0.07	0.0032	9	0.08	0.01	***0.0001***	8	0.072	0.010	8	0.083	0.008	***0.018***
Decay time (s)	9	0.11	0.014	9	0.09	0.01	***0.0004***	8	0.11	0.018	8	0.088	0.009	***0.002***
Rise slope (Ratio/s)	9	2.35	0.64	9	1.27	0.57	***0.0057***	8	2.38	0.79	8	1.39	0.22	***0.005***
Decay slope (Ratio/s)	9	1.50	0.49	9	1.21	0.49	0.25	8	1.68	0.83	8	1.37	0.27	0.25

**Parameter**	**Twitch-4**							**TN-XXL**						
	**Atrium**			**Ventricle**			**p-value (*t*-test)**	**Atrium**			**Ventricle**			**p-value (*t*-test)**
	n	**Average**	**SD**	***n***	**Average**	**SD**		***n***	**Average**	**SD**	***n***	**Average**	**SD**	
Systolic ratio	35	2.16	0.22	35	1.99	0.20	***4.9E-05***	7	1.52	0.14	7	1.35	0.058	***0.021***
Diastolic ratio	35	2.04	0.21	35	1.91	0.20	***9.4E-04***	7	1.47	0.12	7	1.34	0.053	***0.040***
Amplitude	35	0.12	0.039	35	0.081	0.027	***7.3E-07***	7	0.045	0.035	7	0.015	0.011	***0.038***
Heart rate (BPM)	35	217.0	14.5	35	217.1	14.7	0.93	7	223.5	6.4	7	223.7	6.2	0.454
Rise time (s)	35	0.062	0.007	35	0.083	0.006	***5.8E-19***	7	0.070	0.017	7	0.079	0.009	0.165
Decay time (s)	35	0.12	0.013	35	0.089	0.008	***4.3E-18***	7	0.103	0.020	7	0.087	0.014	0.061
Rise slope (Ratio/s)	35	1.98	0.74	35	0.98	0.35	***4.0E-10***	7	0.74	0.67	7	0.200	0.14	***0.049***
Decay slope (Ratio/s)	35	1.03	0.32	35	0.91	0.28	0.064	7	0.42	0.29	7	0.171	0.12	***0.047***

Table S6. Data for Figure 6A. In vivo response to nifedipine measured with Twitch-4.

Chamber	Parameter	Time (h)	Nifedipine 1μM					Nifedipine 10μM					Nifedipine 100μM				
			n	Average	SD	p-value (*t*-test)	Change %	*n*	Average	SD	p-value (*t*-test)	Change %	*n*	Average	SD	p-value (*t*-test)	Change %
**Atrium**	Average ratio	0	5	2.06	0.10	***0.014***	-7.6	6	2.21	0.10	***4.7E-04***	-13.1	5	2.23	0.21	***8.6E-05***	-11.7
		1		1.90	0.18				1.92	0.17				1.97	0.21		
	Amplitude	0	5	0.10	0.05	***0.026***	-30.0	6	0.12	0.02	***1.8E-05***	-66.9					
		1		0.07	0.04				0.04	0.02							
	Heart rate (BPM)	0	5	204.3	13.9	***0.050***	-17.3	6	209.3	16.1	***2.9E-04***	-20.7					
		1		169.0	19.6				166.1	9.2							
	Rise time (s)	0	5	0.07	0.01	0.282	12.5	6	0.06	0.01	0.080	26.1					
		1		0.08	0.01				0.08	0.02							
	Decay time (s)	0	5	0.12	0.01	***0.031***	32.4	6	0.13	0.01	***0.011***	29.4					
		1		0.16	0.03				0.16	0.02							
	Rise slope (Ratio/s)	0	5	1.56	0.83	***0.018***	-33.5	6	1.98	0.50	***2.3E-04***	-72.9					
		1		1.04	0.77				0.54	0.24							
	Decay slope (Ratio/s)	0	5	0.85	0.39	***0.015***	-46.4	6	1.00	0.19	***9.1E-06***	-74.7					
		1		0.46	0.30				0.25	0.11							
**Ventricle**	Average ratio	0	5	1.89	0.13	***0.019***	-5.2	6	2.09	0.13	***0.0074***	-11.5	6	1.97	0.07	***4.4E-04***	-11.6
		1		1.79	0.17				1.85	0.18				1.74	0.13		
	Amplitude	0	5	0.08	0.02	0.520	4.6	6	0.07	0.02	***0.025***	-40.2	6	0.10	0.03	***0.001***	-64.0
		1		0.08	0.02				0.04	0.01				0.04	0.01		
	Heart rate (BPM)	0	5	204.0	13.9	***0.045***	-17.2	6	207.2	16.4	***0.0012***	-20.1	6	215.4	12.3	***0.0044***	-35.9
		1		168.9	18.6				165.6	10.7				138.1	30.9		
	Rise time (s)	0	5	0.09	0.01	0.225	9.9	6	0.086	0.01	0.398	7.0	6	0.08	0.01	0.185	26.0
		1		0.10	0.01				0.092	0.01				0.11	0.03		
	Decay time (s)	0	5	0.09	0.01	***0.029***	44.2	6	0.09	0.01	***0.0011***	48.9	6	0.09	0.01	***0.038***	112.3
		1		0.13	0.03				0.14	0.01				0.19	0.09		
	Rise slope (Ratio/s)	0	5	0.88	0.24	0.114	-7.1	6	0.82	0.17	***0.013***	-44.4	6	1.23	0.42	***0.0051***	-70.2
		1		0.82	0.22				0.45	0.08				0.37	0.13		
	Decay slope (Ratio/s)	0	5	0.85	0.25	***0.011***	-29.4	6	0.76	0.17	***0.0024***	-60.1	6	1.12	0.28	***6.5E-04***	-82.0

No parameters are shown in atrium with 100μM Nifedipine because beating stopped altogether.

Table S7. Data for Figure 6B. Effect of nifedipine on the average ratio measured with Twitch-1, Twitch-2B and TN-XXL biosensors.

**Average ratio**		**Time (h)**	**Nifedipine 1µM**					**Nifedipine 10µM**					**Nifedipine 100µM**				
			n	**Average**	**SD**	**p-value (*t*-test)**	**Change %**	***n***	**Average**	**SD**	**p-value (*t*-test)**	**Change %**	***n***	**Average**	**SD**	**p-value (*t*-test)**	**Change %**
Atrium	Twitch-1	0	4	3.46	0.61	0.104	-19.0	4	4.53	0.32	***2.0E-05***	-52.6	4	4.08	0.42	***8.2E-03***	-48.9
		1		2.80	0.20				2.15	0.30				2.09	0.38		
	Twitch-2B	0	3	4.35	0.61	***3.5E-04***	-33.4	4	5.31	0.30	***3.4E-04***	-55.1	3	4.58	1.41	0.112	-51.2
		1		2.89	0.65				2.39	0.19				2.24	0.20		
	TN-XXL	0	3	1.35	0.16	***0.033***	-10.1	5	1.51	0.18	***0.0033***	-15.7	3	1.67	0.14	0.060	-18.4
		1		1.22	0.14				1.27	0.11				1.36	0.09		
Ventricle	Twitch-1	0	4	3.62	0.53	***0.041***	-19.3	6	4.23	0.70	***0.0109***	-37.5	4	4.03	0.59	***0.012***	-48.5
		1		2.92	0.28				2.64	0.46				2.07	0.21		
	Twitch-2B	0	5	4.58	0.29	***0.002***	-29.3	5	6.56	0.28	***2.8E-04***	-56.8	4	6.07	0.60	***0.002***	-57.0
		1		3.24	0.45				2.83	0.50				2.61	0.46		
	TN-XXL	0	4	1.35	0.06	0.***038***	-7.7	3	1.38	0.06	***0.041***	-13.3	3	1.32	0.03	0.083	-13.5
		1		1.24	0.06				1.20	0.02				1.14	0.07		

Table S8. Data for Figure 6C. Effect of nifedipine on the heart rate and fractional area change in control beating hearts (not treated with PAB) measured by transmitted light.

**Parameter**	**Time (h)**	**Control**			**Nifedipine 5µM**				**Nifedipine 20µM**			
		n	**Average**	**SD**	***n***	**Average**	**SD**	**p-value (*t*-test, vs control)**	***n***	**Average**	**SD**	**p-value (*t*-test, vs control)**
Heart rate (BPM)	0	3	222.5	4.3	4	236.3	15.6	0.2062	3	232.5	7.5	0.1161
	1		242.5	4.3		148.1	7.2	***<0.0001***		150	0	***<0.0001***
	2		250	4.3		163.1	7.2	***<0.0001***		140	18.9	***0.0006***
Fractional area change %	0	3	35.1	5.0	4	34.1	2.2	0.7288	3	44.5	2.9	***0.0479***
	1		41.6	3.7		20.4	5.1	***0.0018***		15.6	7.2	***0.005***
	2		40.9	3.0		17.5	7.5	***0.0041***		13.2	3.6	***0.0005***

Table S9. Data for Figure 7B. In vivo Ca^{2+} kinetics in the heart of Twitch-4-expressing embryos in response to 100 µM propranolol.

Parameter	Atrium						Ventricle					
	**Time (h)**	***n***	**Average**	**SD**	**p-value (*t*-test)**	**Change %**	**Time (h)**	***n***	**Average**	**SD**	**p-value (*t*-test)**	**Change %**
Systolic ratio	0	11	1.80	0.13	***2.7E-05***	-5.9	0	13	1.78	0.12	***3.4E-08***	-6.9
	1		1.69	0.10			1		1.66	0.11		
Diastolic ratio	0	11	1.70	0.12	***1.4E-05***	-5.9	0	13	1.71	0.12	***2.2E-08***	-6.9
	1		1.60	0.08			1		1.59	0.10		
Amplitude	0	11	0.098	0.033	0.301	-5.3	0	13	0.070	0.019	0.116	-6.4
	1		0.093	0.038			1		0.065	0.019		
Heart rate (BPM)	0	11	221.8	13.1	***2.4E-07***	-14.6	0	13	223.4	12.7	***8.0E-09***	-14.4
	1		189.4	15.5			1		191.3	15.0		
Rise time (s)	0	11	0.061	0.006	***0.0425***	15.5	0	13	0.081	0.007	***0.0011***	7.4
	1		0.070	0.013			1		0.087	0.007		
Decay time (s)	0	11	0.117	0.016	***6.8E-06***	21.1	0	13	0.089	0.007	***4.0E-06***	31.6
	1		0.141	0.015			1		0.117	0.017		
Rise slope (Ratio/s)	0	11	1.66	0.59	***0.0466***	-14.2	0	13	0.87	0.20	***0.0092***	-12.9
	1		1.43	0.67			1		0.75	0.22		
Decay slope (Ratio/s)	0	11	0.85	0.26	***4.7E-05***	-21.1	0	13	0.80	0.21	***6.3E-06***	-28.9
	1		0.67	0.28			1		0.57	0.17		

Table S10. Effect of the solvent DMSO (1%) on the kinetics of Ca^{2+} changes in the heart of Twitch-4-expressing embryos.

Parameter	Atrium						Ventricle					
	**Time (h)**	***n***	**Average**	**SD**	**p-value (*t*-test)**	**Change %**	**Time (h)**	***n***	**Average**	**SD**	**p-value (*t*-test)**	**Change %**
Systolic ratio	0	8	1.87	0.11	0.242	-0.8	0	9	1.74	0.11	0.140	-1.2
	1		1.86	0.11			1		1.72	0.11		
Diastolic ratio	0	8	1.79	0.12	0.090	-1.0	0	9	1.67	0.11	0.067	-1.5
	1		1.77	0.12			1		1.65	0.11		
Amplitude	0	8	0.080	0.018	0.378	4.6	0	9	0.069	0.019	0.057	5.1

	1		0.083	0.022			1		0.073	0.019		
Heart rate (BPM)	0	8	221.0	9.6	0.263	0.8	0	9	220.5	9.1	0.155	0.9
	1		222.8	10.2			1		222.6	9.5		
Rise time (s)	0	8	0.064	0.009	0.060	-3.7	0	9	0.081	0.006	0.484	-1.0
	1		0.061	0.009			1		0.080	0.007		
Decay time (s)	0	8	0.13	0.035	0.448	-10.6	0	9	0.091	0.007	0.538	1.2
	1		0.11	0.021			1		0.092	0.008		
Rise slope (Ratio/s)	0	8	1.29	0.38	0.197	10.5	0	9	0.87	0.28	***0.023***	7.6
	1		1.43	0.57			1		0.94	0.31		
Decay slope (Ratio/s)	0	8	0.73	0.08	0.882	0.6	0	9	0.77	0.18	0.158	4.2
	1		0.73	0.10			1		0.80	0.19		

2. SUPPLEMENTARY FIGURES

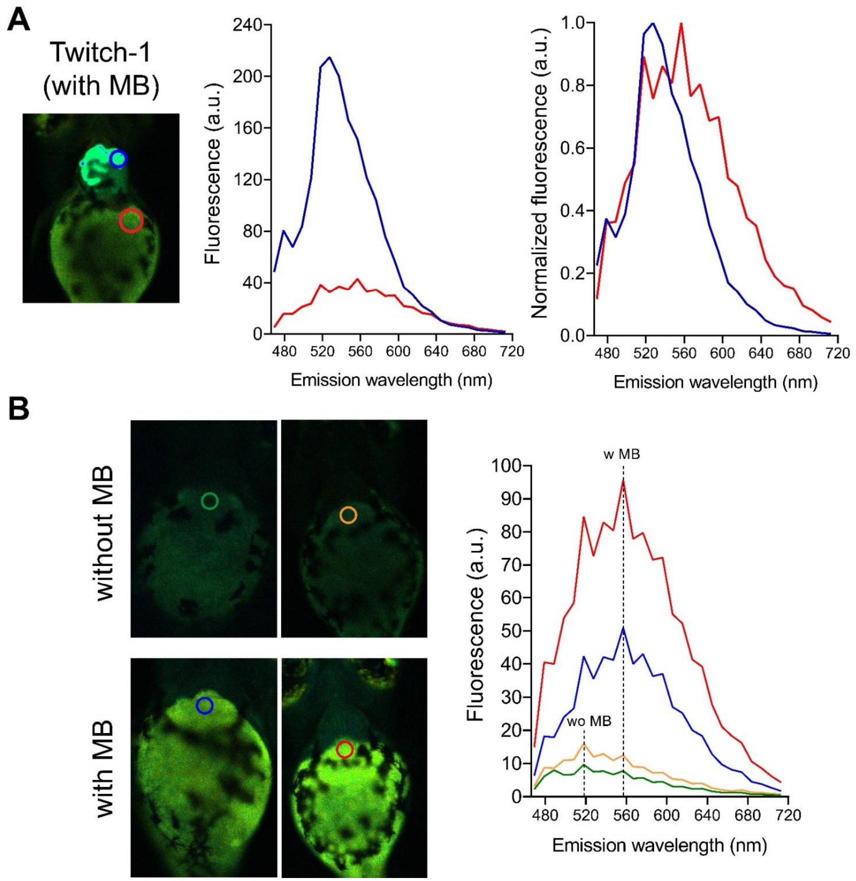

Figure S1. Fluorescence of Twitch-1 compared to autofluorescence of the yolk and effect of methylene blue on yolk autofluorescence in uninjected embryos. A) Wavelength image stacks (x, y, λ) of embryos expressing Twitch-1 in the heart (3 dpf) were obtained with a confocal microscope. The image is a projection of the λ-scan, each spectral plane represented by its color. The graphs show the fluorescence spectra of the heart and of the yolk in the indicated ROIs. The spectra on the right graph were normalized. B) Uninjected embryos (3 dpf) were maintained from day 0 with (w MB) or without methylene blue (wo MB) as indicated. The images are a projection of the λ-scans, each spectral plane represented by its color. The graph shows the fluorescence intensity as a function of the emission wavelength in the indicated ROIs on the image stacks. Methylene blue increased the autofluorescence of the yolk and right-shifted its emission spectra.

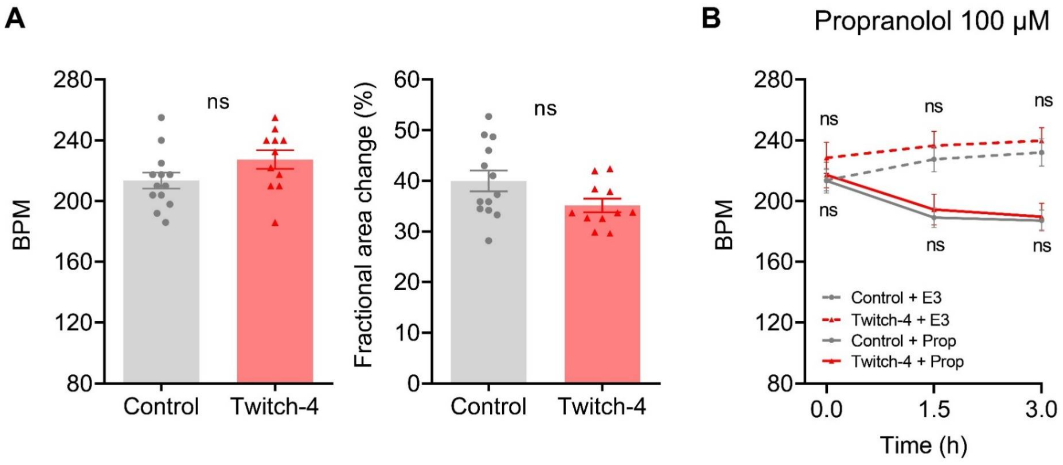

Figure S2. Effects of expression of Twitch-4 on the heart rate (HR), force of contraction and on the effect of propranolol on the HR. A) Effect of expression of Twitch-4 on the HR and on the force of contraction (fractional area change) estimated by transmitted light. Data are shown as the mean ± S.E.M., n = 11 and 13 embryos for the control and Twitch-4 groups, respectively, from two independent experiments. An unpaired Student's t-test was used. B) Effect of propranolol (100 μM) on the HR measured by transmitted light in uninjected control embryos and in embryos expressing Twitch-4 (E3: E3 medium; Prop, propranolol). ns, no significant change was observed between control and Twitch-4 groups for each treatment and time point. An unpaired Student's t-test was used.

3. SUPPLEMENTARY VIDEOS

Video S1. Heart beating in a control embryo (transmitted light).
Video S2. Heart beating in an embryo treated with the myosin inhibitor PAB (transmitted light).
Video S3. Emission ratio of embryo expressing Twitch-1 (treated with PAB).
Video S4. Emission ratio of embryo expressing Twitch-4 (treated with PAB).
Video S5. Emission ratio of embryo expressing FRET control ECFP-16aa-EYFP (treated with PAB).

© 2020 by the authors. Submitted for possible open access publication under the terms and conditions of the Creative Commons Attribution (CC BY) license (http://creativecommons.org/licenses/by/4.0/).
